# Phytochemical Composition, Hepatoprotective, and Antioxidant Activities of *Phyllodium pulchellum* (L.) Desv

**DOI:** 10.3390/molecules23061361

**Published:** 2018-06-05

**Authors:** Ya-Chu Fan, Shi-Jun Yue, Zhong-Long Guo, Lan-Ting Xin, Chao-Yi Wang, Dong-Lin Zhao, Hua-Shi Guan, Chang-Yun Wang

**Affiliations:** 1 Key Laboratory of Marine Drugs, The Ministry of Education of China, School of Medicine and Pharmacy, Ocean University of China, Qingdao 266003, China; fanyachu@163.com (Y.-C.F.); shijun_yue@163.com (S.-J.Y.); 15726227761@163.com (Z.-L.G.); xinlanting1993@163.com (L.-T.X.); chaoyi0411@126.com (C.-Y.W.); zhaodonglin@caas.cn (D.-L.Z.); 2 Laboratory for Marine Drugs and Bioproducts, Qingdao National Laboratory for Marine Science and Technology, Qingdao 266071, China; 3 Marine Agriculture Research Center, Tobacco Research Institute of Chinese Academy of Agricultural Sciences, Qingdao 266101, China

**Keywords:** *Phyllodium pulchellum*, flavonoids, hepatoprotective, antioxidant, HPLC-LTQ-Orbitrap-MS

## Abstract

*Phyllodiumpulchellum* has been traditionally used as a medicinal herb because of its health-promoting effects, such as its hepatoprotective and antioxidant activities. In the present study, the petroleum ether fraction, ethyl acetate fraction, *n*-butanol fraction, and aqueous fraction were successively obtained from the ethanol extract of *P. pulchellum*. Two fractions, ethyl acetate fraction and *n*-butanol fraction, were found to display hepatoprotective and antioxidant activities. Further chemical investigation of the active fractions led to the isolation of its main constituents, including 11 flavonoids (**1**–**11**) and 8 indole alkaloids (**12**–**19**). There were 9 flavonoids (**1**–**9**) that were obtained from the ethyl acetate fraction, and 2 flavonoids (**10** and **11**) and 8 alkaloids (**12**–**19**) from the *n*-butanol fraction. Compounds **1**–**11** and **16**–**19** were isolated for the first time from *P. pulchellum*, and **1**, **2**, **8**, **11**, and **18** were obtained from the genus *Phyllodium* initially. Subsequently, the isolated compounds were evaluated for their in vitro hepatoprotective effects on the human normal hepatocyte cell line L-O2 injured by d-galactosamine and radical scavenging activities against 1,1-diphenyl-2-picrylhydrazyl (DPPH). The flavonoids (−)-epigallocatechin (**5**) and (−)-epicatechin (**6**) exhibited prominent hepatoprotective activities with higher cell viability values (65.53% and 62.40% at 10 μM·mL^−1^, respectively) than the positive control, silymarin (61.85% at 10 μM·mL^−1^). In addition, compared with the positive control of vitamin C (IC_50_: 5.14 μg·mL^−1^), (−)-gallocatechin (**3**) and (−)-epigallocatechin (**5**) exhibited stronger antioxidant activities with IC_50_ values of 3.80 and 3.97 μg·mL^−1^, respectively. Furthermore, the total flavonoids from *P. pulchellum* were characterized using a high-performance liquid chromatography-linear ion trap quadrupole-Orbitrap-mass spectrometry (HPLC-LTQ-Orbitrap-MS). In total, 34 flavonoids were tentatively identified, which had not been previously reported from *P. pulchellum*. In addition, we performed a semi-quantitative analysis of the isolated flavonoids. The contents of compounds **1**–**11** were 3.88, 17.73, 140.35, 41.93, 27.80, 4.34, 0.01, 0.20, 9.67, 795.85, and 5.23 μg·g^−1^, respectively. In conclusion, this study revealed that the flavonoids that were isolated from *P. pulchellum* showed hepatoprotective and antioxidant activities, indicating that, besides alkaloids, the flavonoids should be the potential pharmacodynamic ingredients that are responsible for the hepatoprotective and antioxidant activities of *P. pulchellum*.

## 1. Introduction

*Phyllodium pulchellum* (L.) Desv., once classified as *Desmodium* genus, is a shrub belonging to the *Phyllodium* genus, family Papilionoideae. It is mainly distributed in Southern China [1] and India [2]. As a traditional Chinese medicine, it has been used for the treatment of the enlargement of the liver and spleen, cold fever, malaria, rheumatism bone pains, and swelling [3]. The crude extract of *P. pulchellum* was found to possess antifibrotic [4], antioxidant [5], antitumor [6], antidiarrhea [3], antihypertensive [7], and antiarrhythmic [8] activities. Furthermore, the total alkaloids that were isolated from *P. pulchellum* also exhibited significant antifibrotic activity [9,10,11] and monoamine oxidase inhibitory activity [12].

Over the past five decades, a few chemical constituents were isolated and identified from *P. pulchellum* by various chromatographic, MS, and NMR technologies. There have been 18 alkaloids that have been reported, namely, *N*,*N*-dimethyltryptamine, gramine, 5-hydroxy-*N*,*N*-dimethyltryptamine, 5-hydroxy-*N*-methyltryptamine, 5-methoxy-*N*,*N*-dimethyltryptamine, 5-methoxy-*N*-methyltryptamine, *N*,*N*-dimethyltryptamine oxide, 5-methoxy-*N*,*N*-dimethyltryptamine-oxide, 5-hydroxy-*N*,*N*-dimethyltryptamine-oxide, *N*,*N*,*N*-trimethyltryptamine, 5-methoxy-*N*,*N*,*N*-trimethyl-1*H*-indole-3-ethanaminium, 1-methyl-9*H*-pyrido[3,4-*b*]indol-2-ium, 6-methoxy-1,2-dimethyl-9H-pyrido[3,4-*b*]indol-2-ium, 1,2-dimethyl-1,2,3,4-tetrahydro-*β*-carboline, 6-methoxy-2-methyl-1,2,3,4-tetrahydro-*β*-carboline, 3-indolcarbaldehyde, 3-indolcarbaldehyde, and uridine [7,12,13,14,15]. There have been 6 flavonoids, namely, pulcheloid B, citrusinol, yukovanol, 3,5,2′,4′-tetrahydroxy-2,2″-dimethylpyrano-[5″,6″,7,8]-flavanone, citflavanone, and 8-prenylated 5,7,3′,4′-tetrahydroxy flavanone [6,14,16], that have been reported. There have been 16 phenols that have been found, namely, pulchelstyrenes A–F, 4-hydroxy-2,3-dimethoxybenzaldehyde, *p*-hydroxybenzoic acid, protocatechuic acid, 2-*O*-(3,4-dihydroxybenzoyl)-2,4,6-trihydroxyphenylacetic acid, protocatechuic acid methyl ester, protocatechuic acid ethyl ester, gallic acid ethyl ester, *p*-coumaric acid, caffeic acid ester, and arbutin [6,14,15,16]. There have been 2 glycosides, galactomannan and physcion 1-glycosyl rhamnoside [17,18], that have been reported, as well as 1 lignan derivative methyl piperitol [6], 1 steroid daucosterol [15], and 1 terpene loliolide [15]. Among them, alkaloids have been recognized asing be the active constituents that are responsible for the hepatoprotective activity of this species [7]. The quality control of *P. pulchellum* was established by detecting *N*,*N*-dimethyltryptamine and 5-methoxy-*N*,*N*-dimethyltryptamine with an HPLC-DAD. The results showed that the highest contents of *N*,*N*-dimethyltryptamine and 5-methoxy-*N*,*N*-dimethyltryptamine in the roots of the *P. pulchellum* that were collected in September from Guangxi, China, were 0.106 and 3.260 g × 100 g^−1^, respectively [19]. However, little is known about the bioactivity of other types of compounds from *P. pulchellum*.

This study attempted to investigate the main hepatoprotective and antioxidant ingredients of *P. pulchellum*. Four fractions, namely, the petroleum ether fraction (PPP), ethyl acetate fraction (PPE), *n*-butanol fraction (PPB), and aqueous fraction (PPA), were successively obtained from the ethanol extract of *P. pulchellum*. These four fractions were screened for their hepatoprotective and antioxidant activities. As the PPE and PPB showed the hepatoprotective and antioxidant activities, we further investigated the active principles from the target fractions. Subsequently, the structure elucidation, biological activities, and structure-activity relationships of the isolated compounds were studied and discussed. It was indicated that, besides the alkaloids, the flavonoids could be another potential pharmacodynamic ingredient for driving the hepatoprotective and antioxidant activities of *P. pulchellum*. Because the flavonoids showed good activities, we further investigated the composition of the total flavonoids of *P. pulchellum* using high-performance liquid chromatography-linear ion trap quadrupole-Orbitrap-mass spectrometry (HPLC-LTQ-Orbitrap-MS). It was the first report to characterize the flavonoid compositions of *P. pulchellum* by LC-MS.

## 2. Materials and Methods

### 2.1. Plant Material

The aerial parts of the *P. pulchellum* were collected from Xingning (GPS coordinates: N 23°50′, E 115°30′), Guangdong, China, in July 2014, and were identified by Professor Fengqin Zhou, Shandong University of Chinese Medicine. A voucher specimen (No. PP-201407) was deposited at the Key Laboratory of Marine Drugs, the Ministry of Education of China, Ocean University of China, Qingdao, China.

### 2.2. Reagents

Methanol (MeOH, HPLC grade), ethanol, acetone, petroleum ether (PE), ethyl acetate (EtOAc), *n*-butanol (*n*-BuOH), and dichloromethane (CH_2_Cl_2_) were purchased from Tianjin Siyou Chemical Reagent Co., Ltd. (Tianjin, China). The HPLC-grade acetonitrile was acquired from Fisher Scientific (Fair Lawn, NJ, USA). The formic acid (HPLC grade) was purchased from Sigma Aldrich (St. Louis, MO, USA). The d-Glucose, l-glucose, l-rhamnose, silymarin, dimethyl sulfoxide (DMSO), and 1,1-diphenyl-2-picrylhydrazyl (DPPH) were the products of Sigma (St. Louis, MO, USA). The Dulbecco modified eagle medium (DMEM) was purchased from GIBCO (Carlsbad, CA, USA). The calf serum was purchased from Hangzhou Sijiqing Biological Engineering Materials Co., Ltd. (Hangzhou, China). The phosphate buffer saline (PBS), trypsin-EDTA solution, and penicillin and streptomycin mixture were obtained from Nanjing Kaiji Biotechnology Development Co., Ltd. (Nanjing, China). The human normal hepatocyte cell line L-O2 were purchased from the Cell Resource Center, IBMS, CAMS/PUMC (Beijing, China). The d-galactosamine (d-GalN) was purchased from Wako Co., Ltd. (Tokyo, Japan). The 3-(4,5-dimethyl-2-thiazolyl)-2,5-diphenyl-2-*H*-tetrazolium bromide (MTT) was purchased from Biosharp (Hefei, China). The vitamin C was a product of the Sinopharm Group Chemical Reagent Co., Ltd. (Beijing, China). The rutin was purchased from the National Institutes for Food and Drug Control (Beijing, China).

### 2.3. General Experimental Procedures

Optical rotations were measured on a JASCO P-1020 digital polarimeter (JASCO Corporation, Tokyo, Japan). The IR spectra were recorded on a Nicolet-Nexus-470 spectrometer (Nicolet Corp., Madison, WI, USA). The NMR spectra were recorded on an Agilent DD2 500 MHz NMR spectrometer (500 MHz for ^1^H and 125 MHz for ^13^C), using TMS as an internal standard (Agilent Technologies, Santa Clara, CA, USA). The ESI-MS spectra were obtained from a Micromass Q-TOF spectrometer (Waters Corp., Milford, MA, USA). The HPLC-LTQ-Orbitrap-MS analysis was performed on an Agilent series 1290 Infinity HPLC instrument (Agilent, Technologies, Santa Clara, CA, USA), coupled with an LTQ/Orbitrap mass spectrometer (Thermo Scientific, Bremen, Germany) that was equipped with an electrospray (ESI) interface. The semipreparative HPLC was performed using a Hitachi prep-HPLC system that was coupled with a Hitachi L-2455 diode array detector (Hitachi Corp., Tokyo, Japan) and a Kromasil C-18 preparative HPLC column (250 mm × 10 mm, 5 mm) (Eka Nobel, Bohus, Sweden). Silica gel (Qing Dao Hai Yang Chemical Group Co., Qingdao, China; 200–300 mesh) and Sephadex LH-20 (Amersham Biosciences, Inc., Piscataway, NJ, USA) were used for column chromatography. The pre-coated silica gel plates (Yantai Zifu Chemical Group Co., Yantai, China; G60 and F-254) were used for thin-layer chromatography.

### 2.4. Extraction and Isolation

The dried aerial parts of the *P. pulchellum* (8.0 kg) were consecutively extracted three times by reflux, with 95% ethanol at 80 °C for 3 h. The combined ethanol layer was lyophilized in order to obtain the ethanol extract (950 g). This dried residue was mixed with distilled water (1000 mL) and successively partitioned three times with the same volume of petroleum ether (PE), EtOAc, and *n*-butanol (*n*-BuOH). The respective fractions were dried under reduced pressure and lyophilized to yield four fractions, namely, PPP (60.0 g), PPE (50.0 g), PPB (30.0 g), and PPA (20.0 g). The above fractions were stored at −20 °C.

The PPE (40.0 g) was purified using a vacuum liquid chromatography (VLC) on silica gel, using a step gradient elution with EtOAc/PE (0−100%), so as to afford six fractions (Fractions 1–6). Fraction 3 was applied to a silica gel column chromatography with CH_2_Cl_2_/MeOH (50:1 to 0:100), in order to provide 12 subfractions (subfrations 3.1–3.12). Subfraction 3.5 was isolated using a Sephadex LH-20 column with mixtures of CH_2_Cl_2_/MeOH (1:1) and preparative HPLC with 50% MeOH to produce compound 8 (5.7 mg). Subfraction 3.7 was subjected to a silica gel column with PE/CH_2_Cl_2_/MeOH (2:1:1) in order to yield compound 9 (30.0 mg). Subfraction 3.12 was purified using a Sephadex LH-20 column with CH_2_Cl_2_/MeOH (1:1), and was finally purified using preparative HPLC with 30% MeOH to obtain compound 7 (13.8 mg). Fraction 4 was subjected to a silica gel column with CH_2_Cl_2_/MeOH (50:1 to 1:100) to afford six subfractions (subfraction 4.1‒4.6). Subfraction 4.2 was applied to a Sephadex LH-20 column with MeOH, followed by an octadecylsilyl (ODS) column chromatography with MeOH/H_2_O (30:70 to 100:0) and preparative HPLC with 30% MeOH, so as to obtain compounds 1 (53.0 mg) and 2 (12.5 mg). Subfraction 4.6 was separated under the same chromatography conditions as Subfraction 4.2, to afford compounds 3 (7.7 mg), 4 (13.4 mg), 5 (11.2 mg), and 6 (51.0 mg).

The PPB (20.0 g) was separated into five fractions (fraction 1–5) by VLC, using a step gradient elution with MeOH/CH_2_Cl_2_ (0−100%). Fraction 3 was subjected to a silica gel column with CH_2_Cl_2_/MeOH/NH_3_·H_2_O (30:1 to 0:100), to afford nine subfractions (subfraction 3.1–3.9). Subfraction 3.3 was applied to a Sephadex LH-20 column with MeOH and a preparation of thin-layer chromatography in order to obtain compounds 12 (45.0 mg) and 14 (13.0 mg). Subfraction 3.5 was separated under the same chromatography conditions as subfraction 3.3, to give compounds 13 (48.0 mg) and 15 (24.0 mg). Subfraction 3.9 was first subjected to a Sephadex LH-20 column with MeOH and an ODS column with MeOH/H_2_O (30:70 to 100:0), and was further purified on preparative HPLC with 45% MeOH, to yield compounds 16 (10.0 mg) and 17 (12.5 mg). Fraction 5 was subjected to a silica gel column using CH_2_Cl_2_/MeOH (50:1 to 0:100) to afford two subfractions (subfractions 5.1 and 5.2). Subfraction 5.1 was fractionated under the same chromatography conditions as subfraction 3.9, to provide compounds 10 (12.6 mg) and 11 (6.6 mg). Similarly, subfraction 5.2 was first subjected to a Sephadex LH-20 column with MeOH, and was then separated by an ODS column with MeOH/H_2_O (30:70 to 100:0), and was further purified on preparative HPLC with 20% MeOH to form compounds 18 (5.7 mg) and 19 (2.7 mg).

### 2.5. Hepatoprotective Activity Assay

The hepatoprotective effects were tested on human normal hepatocyte cell line L-O2 injured by d-GalN using MTT method, as reported previously [20]. In this assay, the cell viability was measured so as to reflect the hepatoprotective activity. The cells were prepared into a 6.0 × 10^4^·mL^−1^ cell suspension with a DMEM medium containing 10% fetal bovine serum, penicillin (100 U·mL^−1^), and streptomycin (100 mg·mL^−1^), which were seeded in 96-well culture medium. After incubation for 24 h in an incubator with 5% CO_2_ at 37 °C, 100 μL test samples were added. After incubation for 1 h, 37 mM d-GalN was added and incubated for 24 h. The incubation solution was discarded, and 100 μL MTT solution (0.5 mg·mL^−1^) was added to each well, followed by incubation at 37 °C for 4 h. The supernatant was discarded and 150 μL DMSO was added to each well so as to dissolve the fomazan particles. After a mild shaking, the optic density (OD) value was measured at the detection wavelength of 490 nm. Silymarin (purity > 98%) was used as a positive control. The blank control group was established at the same time, with 37 mM d-GalN.

### 2.6. DPPH Radical-Scavenging Activity Assay

Radical reducing effects against DPPH were tested according to a previously described method [21]. The DPPH radical-scavenging was evaluated by comparing the percentage inhibition of the DPPH radicals. Specifically, for the fractions from *P. pulchellum*, each fraction of PPP, PPE, PPB, and PPA was dissolved in DMSO, and diluted into 156.25, 78.13, 39.06, 19.5, and 9.75 µg·mL^–1^, respectively. For the isolated compounds, each compound was dissolved in DMSO, and were diluted into 200, 100, 50, 25, and 12.5 µg·mL^–1^. The DPPH solution was prepared with anhydrous ethanol to get the concentration of 0.05 mg·mL^–1^. Each of the test samples were added to the DPPH solution 100 μL. After 30 min of dark reaction, the absorbance was measured at 517 nm. DPPH radical reducing activity of the test sample was expressed as *I* = [(*A*_0_/*A*_X_)/*A*_0_] × 100%, where *A*_0_ was the absorbance of the blank control reaction and *A*_X_ was the absorbance in the presence of the sample. The sample concentration, providing 50% inhibition (IC_50_), was calculated from the regression equation that was prepared from the concentration of the samples and the inhibition percentage. Vitamin C (purity > 99.7%) was used as a positive control.

### 2.7. HPLC-LTQ-Orbitrap-MS Analysis

The total flavonoids extract of *P. pulchellum* were obtained by ultrasonic extraction procedure. The ultrasonic extraction conditions were investigated based on the quantitative research of the total flavonoids using UV–Vis spectrophotometry from our previous experiment (data not published). Specifically, the dried *P. pulchellum* (5 g) was extracted with ultrasonic in small eggplant-type bottle with 71% ethanol (150 mL) at 80 °C for 1 h. The extract was evaporated in a rotary evaporator at 40 °C to dryness, and the residue was dissolved in 5 mL of 71% ethanol. This sample was filtered through a 0.22 μm syringe filter before subjected to HPLC-LTQ-Orbitrap-MS. There were 8 compounds that were isolated from *P. pulchellum* that were used as reference standards, including (−)-gallocatechin (compound **3**), (+)-catechin (compound **4**), (−)-epicatechin (compound **6**), dihydroquercetin (compound **7**), (+)-dihydrokaempferol (compound **8**) quercetin (compound **9**), rutin (compound **10**), and quercetin-3-*O*-*α*-l-rhamnopyranoside-(1→6)-*β*-d-galactopyranosyl (compound **11**).

An Agilent series 1290 Infinity HPLC instrument coupled with an LTQ/Orbitrap mass spectrometer equipped with an electrospray (ESI) interface was performed to analyze the total flavonoids extract. The chromatography conditions were as follows: a Kromasil C-18 analytical HPLC column (250 mm × 4.6 mm, I.D., 5 μm) was used; the flow rate was 1.0 mL·min^−1^; the sample injection volume was 10 μL; the column temperature was 30 °C; and the diode-array detector (DAD) scanned from 190 to 400 nm, and the samples were detected at 254 nm. The gradient profile of mobile phase A (acetonitrile) and mobile phase B (0.1% formic acid-water) was as follows: 0–10 min, 5–10% A; 10–20 min, 10–20% A; 20–30 min, 20–50% A; 30–50 min, 50–70% A; 50–60 min, 70–80% A; and 60–65 min, 80–100% A. The LTQ-Orbitrap-MS operating parameters were as follows: drying gas, high-purity nitrogen (N_2_); capillary temperature, 350 °C; source voltage, 3000 V; sheath gas flow, 40 arb; aux gas flow, 10 arb; and tube lens, −100 V. Each sample was analyzed in both positive and negative modes so as to provide abundant information for structural identification. The mass spectra were recorded across the range of *m*/*z* 100–1500, with accurate mass measurement of all of the mass peaks. Accurate mass measurements of each peak from the total ion chromatogram (TIC) were obtained by means of ESI source. All of the data were processed using Xcalibur software version 3.0 (Thermo Fisher Scientific, San Jose, CA, USA).

The quantification of the isolated flavonoids was carried out by high performance liquid chromatography using a semi-quantitative analysis method. Rutin (purity > 92.6%) was used as a standard. The samples and standard were dissolved in ethanol (HPLC grade) and filtered using 0.22 μm sterile Millex filters before injection. Aliquots of 10 μL were injected into the HPLC system.

## 3. Results and Discussion

### 3.1. Hepatoprotective and Antioxidant Activities of Organic Fractions

Four fractions, PPP, PPE, PPB, and PPA, were obtained and evaluated for their hepatoprotective and antioxidant activities. Among them, PPE and PPB showed better hepatoprotective activities against human normal hepatocyte cell line L-O2 injured by d-GalN, with the cell viability value of 64.09% and 57.25% at 50 μM·mL^−1^, respectively, while the PPA was 51.58% and the PPP was 49.47% at 50 μM·mL^−1^. Meanwhile, PPE and PPB showed stronger antioxidant activities for being able to scavenge DPPH radicals with the IC_50_ values of 36.1 and 64.0 µg·mL^−1^, respectively, compared with PPP with the IC_50_ values of 90.9 µg·mL^−1^ and 264.1 µg·mL^−1^, respectively.

### 3.2. Structure Characterization of the Isolated Compounds from Ethyl Acetate Fraction (PPE) and n-Butanol Fraction (PPB)

**Figure 1:**
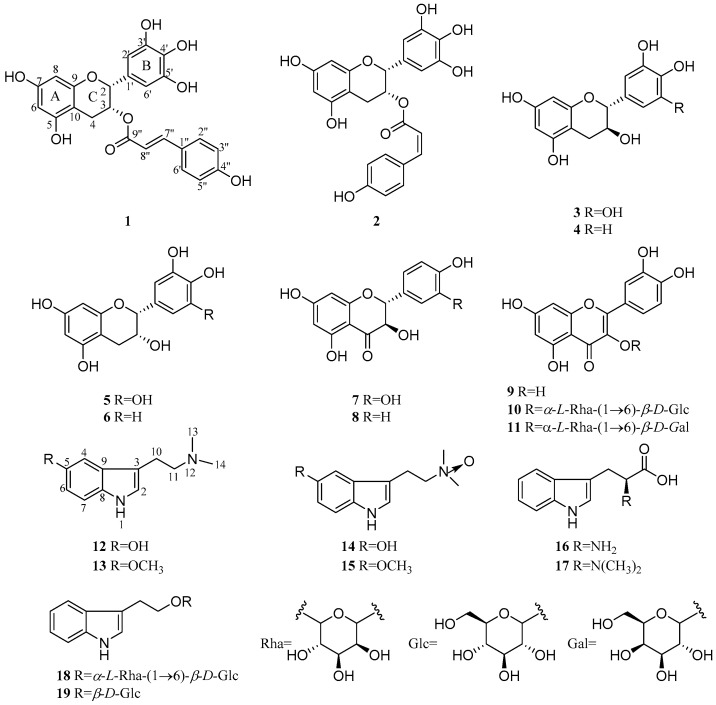
Structures of compounds **1**–**19**, isolated from *P. pulchellum*.

Nineteen compounds (compounds **1**−**19**) were isolated from PPE and PPB, of which 9 flavonoids (flavonoids **1**–**9**) were obtained from PPE, and 2 flavonoids (flavonoids **10** and **11**) and 8 alkaloids (alkaloids **12**–**19**) from PPB (Figure 1). Compounds **1**−**11** were flavonoids with different substitution patterns. Compound **1** was isolated as a light brown powder and was assigned a molecular formula of C_24_H_20_O_9_ on the basis of its NMR and ESI-MS data, which indicated 15 degrees of unsaturation. The ^1^H-NMR spectrum of compound **1** displayed signals for 2 olefinic protons at *δ*_H_ 7.47 (d, *J* = 15.9 Hz) and 6.23 (d, *J* = 15.9 Hz), 5 aromatic protons at *δ*_H_ 5.95‒6.75, 2 methines at *δ*_H_ 5.45 (m) and 4.93 (br s), and 1 methylene at *δ*_H_ 2.96 (dd, *J* = 17.3, 4.6 Hz) and 2.84 (dd, *J* = 17.3, 2.0 Hz). The ^13^C-NMR and DEPT spectra of compound **1** indicated the presence of 24 carbons, including 1 carbonyl carbon at *δ*_C_ 168.6, 18 aromatic carbons at *δ*_C_ 95.8‒157.8, 2 olefinic carbons at *δ*_C_ 133.7 and 115.1, 2 methines at *δ*_C_ 78.4 and 69.8, and 1 methylene at *δ*_C_ 26.7. These data suggested that compound **1** comprised a flavan skeleton with a 3,5,7,3′,4′,5′-hexasubstitution pattern, and was identified as (−)-epigallocatechin 3-*O*-(*E*)-*p*-coumaroate, based on its NMR data and rotation data, $\alpha_{D}^{22}$: −204.5 (MeOH) [22]. The other flavonoids were also determined based on their NMR and ESI-MS data, as (−)-epigallocatechin 3-*O*-(*Z*)-*p*-coumaroate (flavonoid 2) [23], (−)-gallocatechin (flavonoid 3) [24], (+)-catechin (flavonoid 4) [22], (−)-epigallocatechin (flavonoid 5) [25], (−)-epicatechin (flavonoid 6) [22], dihydroquercetin (flavonoid 7) [26], (+)-dihydrokaempferol (flavonoid 8) [27], quercetin (flavonoid 9) [28], rutin (flavonoid 10) [29], and quercetin-3-*O*-*α*-l-rhamnopyranosyl-(1→6)-*β*-d-galactopyranoside (flavonoid 11) [30], respectively (see Supporting Information). All of the flavonoids 1–11 were isolated from *P. pulchellum* for the first time, and flavonoids **1**, **2**, **8**, and **11** were obtained from the genus *Phyllodium* initially.

Compounds **12**−**19** were a series of indole alkaloids. Compound **12** was obtained as a colorless crystal. Its molecular formula was determined to be C_12_H_16_N_2_O (six degrees of unsaturation), based on its ^1^H- and ^13^C-NMR spectra, combined with the ESI-MS data. The ^1^H-NMR spectrum displayed signals for 1 indole nitrogen proton at *δ*_H_ 9.82 (s), 1 olefinic proton at *δ*_H_ 6.33 (s), 3 aromatic protons at *δ*_H_ 6.44 (d, *J* = 8.6 Hz), 6.15 (d, *J* = 2.0 Hz), and 5.93 (dd, *J* = 8.6, 2.0 Hz), 2 methylenes at *δ*_H_ 2.05 (2H, m) and 1.82 (2H, m), and 2 methyl groups at *δ*_H_ 1.54 (s). The ^13^C-NMR and DEPT spectra of compound 12 indicated the presence of 12 carbons, including 6 aromatic carbons at *δ*_C_ 150.2, 130.9, 128.0, 111.7, 111.3, and 102.3; 2 olefinic carbons at *δ*_C_ 123.0 and 111.5; 2 methylenes at *δ*_C_ 60.0 and 23.2; and 2 methyl groups at *δ*_C_ 45.1 and 45.1. Based on the above analysis, compound **12** was identified as 5-hydroxy-*N*,*N*-dimethyltryptamine, which was identical to the reported data [31]. Subsequently, compounds 13−19 were identified as 5-methoxy-*N*,*N*-dimethyltryptamine (compound **13**) [32], 5-hydroxy-*N*,*N*-dimethyltryptamine-oxide (compound **14**) [33], 5-methoxy-*N*,*N*-dimethyltryptamine-oxide (compound **15**) [13], L-tryptophan (compound **16**) [34], *N*,*N*-dimethyl-l-tryptophan (compound **17**) [35], 2-(indol-3-yl)ethyl-*α*-l-rhamnopyranosyl-(1→6)-*β*-d-glucopyranoside (compound **18**) [36], and 2-(indol-3-yl)ethyl-*β*-d-glucopyranoside (compound **19**) [37] (see Supporting Information). Alkaloids **16**–**19** were isolated from *P. pulchellum* for the first time, and alkaloid 18 was obtained from the genus *Phyllodium* initially.

### 3.3. Hepatoprotective Activity of the Isolated Compounds

All of the isolated compounds **1**−**19** were tested for their hepatoprotective activities against the human normal hepatocyte cell line L-O2 injured by d-GalN. The results indicated that flavonoids **5** and **6** exhibited observably hepatoprotective activity, with higher cell viability values (65.53% and 62.40% at 10 μM·mL^−1^, respectively) than the positive control, silymarin (61.85% at 10 μM·mL^−1^). Interestingly, alkaloid **18** was also found to show hepatoprotective activity, with the cell viability value of 60.72% at 10 μM·mL^−1^, which was close to the positive control. According to the above results, it could be presumed that *P. pulchellum* displaying hepatoprotective activity might have been as a result of the presence of active compounds, mainly in the PPE and PPB fractions, such as compounds **5**, **6**, and **18**.

A literature survey revealed that the flavonoids that were isolated from *P. pulchellum*, such as yukovanol and 8-prenylated 5,7,3′,4′-tetrahydroxy flavanone, could have inhibited the proliferation of the activated hepatic stellate cells (HSC-T6 cells), in vitro at 10 μM·L^−1^, with cell viability values of 54% and 42%, respectively [14]. In addition, flavonoid pulcheloid B was reported to exhibit potent inhibitory activity in vitro against the proliferation of acetaldehyde-stimulated HSC-T6 cells, with the IC_50_ value of 7.6 mM [16]. Our study further proved that flavonoids had the hepatoprotective activity, which indicated that, besides the alkaloids, the flavonoids might have also been the potential pharmacodynamic ingredients that were responsible for the hepatoprotective activity of *P. pulchellum*.

### 3.4. Antioxidant Activity of the Isolated Compounds

A literature survey revealed that the hepatoprotective activity might have been related to antioxidant activity [38,39]. In the present study, the isolated compounds **1**−**19** were further tested for their antioxidant activities against DPPH radicals. Only the flavonoids (−)-gallocatechin (compound **3**) and (−)-epigallocatechin (compound **5**) displayed strong antioxidant activities with the IC_50_ values of 3.8 and 4.0 μg·mL^−1^, respectively, which were more potent than the positive control, vitamin C (IC_50_: 5.1 μg·mL^−1^) (Table 1). Our results showed that flavonoids not only exhibited prominent hepatoprotective activity, but that they also displayed strong antioxidant activity. By comparison of the activities of flavonoids **3**–**6**, we found that flavonoids **3** and **5** showed stronger activity than flavonoids 4 and 6, respectively. Furthermore, compound **7** (IC_50_: 47.5 μg·mL^−1^) displayed a stronger activity than that of compound 8 (IC_50_: >300 μg·mL^−1^). These results could have demonstrated that hydroxyl at C-5′ might have played an important role in antioxidant activity, which was consistent with previous reports [40,41]. Of flavonoids **1**, **2**, and **5**, compound **5** with a hydroxyl substitution at C-3 demonstrated higher activity, which indicated that the hydroxyl group at C-3 had a positive contribution to improving the activity rather than the *p*-hydroxy-cinnamic acid substitution. Additionally, compared with compound **9**, the glycoside substitutions of compounds **10** and **11** reduced the activity. Based on the above results, it could have been inferred that the extracts with better antioxidant activity might have been as a result of the active compounds contained in PPE fraction, such as flavonoids **1**–**7** and **9**.

There was no report regarding the antioxidant activity of the flavonoids from *P. pulchellum*. Interestingly, the flavonoids that were isolated from the ethanol extract of the *Distylium racemosum* branches, including compounds **3**−**6** and **9**, which were the same as our study, were also evaluated for their antioxidant activities [42]. Particularly, compounds **3**, **5**, and **9** were reported for their radical scavenging activities toward DPPH, with the IC_50_ values of 6.7, 62, and 65.3 μg·mL^−1^, respectively [42], which were weaker than our test. While compounds 4 and 6 were found to display DPPH radical scavenging activities with the IC_50_ values of 7.2 and 6.3 μg·mL^−1^, respectively [42], which were stronger than our study. Thus, it was necessary to carry out the in vivo antioxidant experiment of the isolated compounds.

### 3.5. HPLC-LTQ-Orbitrap-MS Analysis of Total Flavonoids

**Figure 2:**
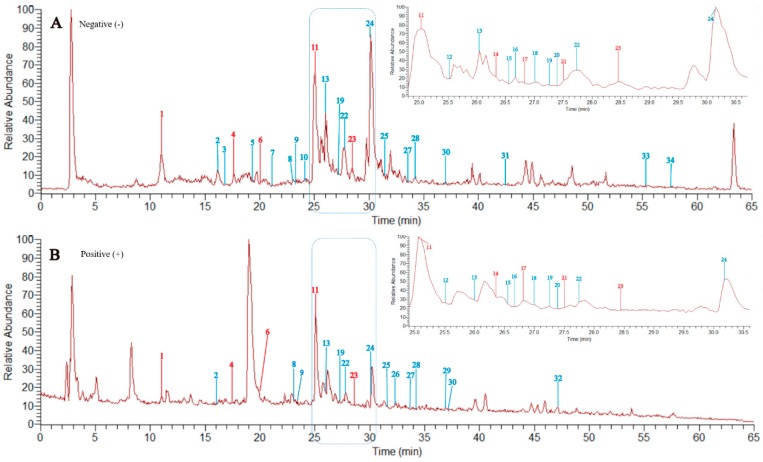
The total ion chromatograms (TIC) of the total flavonoids of *P. pulchellum* by high-performance liquid chromatography-linear ion trap quadrupole-Orbitrap-mass spectrometry (HPLC-LTQ-Orbitrap-MS) in negative ion mode (**A**) and positive ion mode (**B**). The compounds that are confirmed with the isolated reference compounds are marked in red.

To characterize the flavonoids in *P. pulchellum*, a HPLC-LTQ-Orbitrap-MS method was established. The total ion chromatograms (TIC) in negative ion mode (A) and positive ion mode (B) are displayed in Figure 2. Most of the constituents were well separated under the gradient elution condition, with high resolution and good sensitivity.

A total of 34 flavonoids, including 11 isolated flavonoids (flavonoids **1**−**11**), were identified or tentatively characterized using our established analysis method (Figure 2 and Table 2). Among them, 8 compounds were confirmed with the isolated flavonoids as references, including (−)-gallocatechin (compound **3**), (+)-catechin (compound **4**), (−)-epicatechin (compound **6**), dihydroquercetin (compound **7**), (+)-dihydrokaempferol (compound **8**) quercetin (compound **9**), rutin (compound **10**), and quercetin-3-*O*-*α*-l-rhamnopyranoside-(1→6)-*β*-d-galactopyranosyl (compound **11**). The structures of 23 other flavonoids were tentatively characterized based on their retention times, HR-ESIMS data, and fragment ions, referring to databases (representative databases: SciFinder and KNApSAcK Core System) and literatures [43,44,45,46]. All of the identified 34 flavonoids had not been reported previously from *P. pulchellum*. These flavonoids that were characterized by HPLC-LTQ-Orbitrap-MS included 6 flavones (peaks 10, 15, 16, 19, 27, and 33), 11 flavonols (peaks 8, 9,11, 12, 13, 17, 20, 23, 24, 28, and 32), 6 flavan-3-ols (peak 1, 2, 4, 6, 18, and 22), 3 isoflavones (peaks 25, 29, and 31), 2 chalcones (peaks 5 and 26), 3 flavanonols (peak 7, 14, and 21), 1 dihydroflavone (peak 3), 1 flavan-3,4-diols (peak 30), and 1 xanthone (peak 34). To the best of our knowledge, it was the first time that the chemical constituents of flavonoids in *P. pulchellum* had been thoroughly and systematically investigate using HPLC-LTQ-Orbitrap-MS analysis, which would have provided a basis for further study of *P. pulchellum*, such as its metabonomics.

In addition, we performed a semi-quantitative analysis of the isolated flavonoids. The contents of compounds **1**‒**11** were 3.88, 17.73, 140.35, 41.93, 27.80, 4.34, 0.01, 0.20, 9.67, 795.85, and 5.23 μg·g^−1^, respectively. In our previous study, the quantitative investigation gave a total flavonoids content of 24.213 mg·g^−1^ of *P. pulchellum* using UV–Vis spectrophotometry (data not published).

## 4. Conclusions

In summary, we investigated the pharmacodynamic ingredients of *P. pulchellum*, focusing on the flavonoids and their hepatoprotective and antioxidant activities. A phytochemical investigation of the active fractions of PPE and PPB from the ethanol extract of *P. pulchellum* led to the isolation of 11 flavonoids (flavonoids **1**−**11**). The semi-quantification by HPLC showed that *P. pulchellum* might have possessed large quantities of flavonoids. Besides the isolated flavonoids, further investigation on the total flavonoids of *P. pulchellum* by HPLC-LTQ-Orbitrap-MS led to the discovery of 23 other flavonoids. All of the characterized 34 flavonoids were reported from *P. pulchellum* for the first time. These findings not only confirmed the abundant flavonoids in *P. pulchellum*, but also enriched the chemical composition library of this species.

In our study, the isolated flavonoids were found to exhibit prominent hepatoprotective and antioxidant activities, which was suggestive of the potential pharmacodynamic components of *P. pulchellum*. Specifically, flavonoids 5 and 6 exhibited observably hepatoprotective activity, with higher cell viability values than the positive control, silymarin. In addition, in our previous study, flavonoids **1**–**3**, **5**, **7**, and **9** showed DNA topoisomerase I (Topo I) inhibitory activity at a concentration of 100 μM. Particularly flavonoids **1** and **2** still exhibited potent inhibitory activity, even at 5 μM [47]. In general, the flavonoids showed significant biological activities, which should be important pharmacodynamic ingredients of *P. pulchellum*. Further investigation of flavonoids and alkaloids were suggested in order to explore the effects and mechanisms of thepharmacodynamic components from *P. pulchellum* in more detail.

## Figures and Tables

**Table 1:** 1,1-Diphenyl-2-picrylhydrazyl (DPPH)-scavenging activity of compounds **1**–**19** from *P. pulchellum* (*n* = 5).

Compounds	DPPH/IC_50_ (μg·mL^−1^)	Compounds	DPPH/IC_50_ (μg·mL^−1^)
**1**	36.1 ± 2.1	**11**	>300
**2**	33.5 ± 1.3	**12**	>300
**3**	3.8 ± 0.14	**13**	>300
**4**	32.8 ± 0.85	**14**	>300
**5**	4.0 ± 0.09	**15**	>300
**6**	29.0 ± 1.15	**16**	>300
**7**	47.5 ± 2.7	**17**	>300
**8**	>300	**18**	>300
**9**	35.2 ± 2.6	**19**	>300
**10**	>300	Vitamin C	5.1 ± 0.09

**Table 2:** Identification of 34 flavonoids in the ethanol extract of *P. pulchellum* by high-performance liquid chromatography-linear ion trap quadrupole-Orbitrap-mass spectrometry (HPLC-LTQ-Orbitrap-MS).

No.	Rt. (min)	Identification	Formula	Negative Ion (*m*/*z*)		Positive Ion (*m*/*z*)	
				Quasi-Molecular	MS/MS (*m*/*z*)	Quasi-Molecular	MS/MS (*m*/*z*)
1^a^	11.02	(−)-Gallocatechin (compound 3)	C_15_H_14_O_7_	305.0622 [M − H]^−^	287 [M − H − H_2_O]^−^; 261 [M − H − H_2_O − O^2−^]^−^; 221 [M − H − A]^−^; 179 [M − H − B]^−^; 137 [M − H − C_8_H_8_O_4_]^−^	307.0806 [M + H]^+^	289; 181; 139
2	16.13	(−)-Epigallocatechin (compound 5)	C_15_H_14_O_7_	305.0661 [M − H]^−^	n.a.	307.0825 [M + H]^+^	289; 181; 139
3	16.85	5-Hydroxyl liquiritin	C_21_H_22_O_10_	433.2018 [M − H]^−^	387 [M − H − CO − H_2_O]^−^; 353 [M − H − A]^−^; 293; 271 [M − H − C_6_H_11_O_5_]^−^	n.a.	n.a.
4^a^	17.52	(+)-Catechin (compound 4)	C_15_H_14_O_6_	289.0674 [M − H]^−^	271 [M − H − H_2_O]^−^ 245 [M − H − CO_2_]^−^; 205 [M − H − A]^−^; 179 [M − H − C_6_H_6_O_2_]^−^;	291.0878 [M + H]^+^	273; 246; 165; 139
5	19.41	Coreopsin	C_21_H_22_O_10_	433.2014 [M − H]^−^	415 [M − H − H_2_O]^−^; 397 [M − H − H_2_O]^−^; 297 [M − H − C_7_H_5_O_3_]^−^; 161 [glc]^−^	n.a.	n.a.
6^a^	20.80	(−)-Epicatechin (compound 6)	C_15_H_14_O_6_	289.0675 [M − H]^−^	245 [M − H − CO_2_]^−^; 205 [M − H − A]^−^; 179 [M − H − C_6_H_6_O_2_]^−^;	291.0874 [M + H]^+^	n.a.
7	21.08	Dihydrokaempferol-7-*O*-*β*-d-glucoside	C_21_H_22_O_11_	449.1023 [M − H]^−^	287 [M − H − glc]^−^; 267 [M − H − glc − H_2_O]^−^; 259 [M − H − glc − CO]^−^	n.a.	n.a.
8	23.05	Gossypetin 7-rhamnoside-8-glucoside	C_27_H_30_O_17_	625.1320 [M − H]^−^	316 [M − H − glc − rha]^−^; 271 [M − H − glc − rha – OH − CO]^−^	627.1585 [M + H]^+^	481; 319
9	23.39	Quercetin-7-*O*-glucopyranoside	C_21_H_20_O_12_	463.0815 [M − H]^−^	301 [M − H − glc]^−^	465.1043 [M + H]^+^	303
10	24.23	Viscidulin II 2′-*O*-glucoside	C_23_H_26_O_12_	493.1289 [M − H]^−^	331 [M − H − glc]^−^; 313 [M − H − C_6_H_12_O_6_]^−^; 271 [M − H − glc − OCH_3_]^−^	n.a.	n.a.
11^a^	25.06	Rutin (compound 10)	C_27_H_30_O_16_	609.1379 [M − H]^−^	301 [M − H − C_12_H_20_O_9_]^−^	611.1638 [M + H]^+^	303
12	25.54	Morin-7-*O*-glucopyranoside	C_21_H_20_O_12_	463.0891 [M − H]^−^	301.0454 [M − H − glc]^−^	465.1047 [M + H]^+^	303
13	26.04	Kaempferol 3-*O*-rutinoside	C_27_H_30_O_15_	593.1428 [M − H]^−^	327 [M − H − C_12_H_20_O_10_]^−^; 285 [M − H − rutinoside]^−^	595.1683 [M + H]^+^	449; 287
14^a^	26.33	Dihydroquercetin (compound 7)	C_15_H_12_O_7_	303.0462 [M − H]^−^	285 [M − H − H_2_O]^−^; 259 [M − H − CO_2_]^−^; 177 [M − H − C_8_H_4_O_6_]^−^	305.0673 [M + H]^+^	n.a.
15	26.55	Luteolin 7-*O*-rutinoside	C_27_H_30_O_15_	593.1425 [M − H]^−^	285 [M − H − rutinoside]^−^	595.1456 [M + H]^+^	449; 287
16	26.67	Kaempferol-7-*O*-glucoside	C_21_H_20_O_11_	447.0870 [M − H]^−^	285 [M − H − glc]^−^	449.1093 [M + H]^+^	287; 172
17^a^	26.83	Quercetin-3-*O*-*α*-l- rhamnopyranoside-(1→6)- *β*-d-galactopyranosyl (compound 11)	C_27_H_30_O_16_	609.1372 [M − H]^−^	301 [M − H − C_12_H_20_O_9_]^−^	611.1627 [M + H]^+^	n.a.
18	27.00	(−)-Epigallocatechin 3-*O*-(*E*)-*p*-coumaroate (compound 1)	C_24_H_20_O_9_	451.0968 [M − H]^−^	433 [M − H − H_2_O]^−^; 357 [M − H − C_5_H_3_O_2_]^−^; 341; 311; 217	453.1194 [M + H]^+^	n.a.
19	27.28	5,7,2-Trihydroxy-6-methoxyflavone 7-*O*-*β*-d-glucoside	C_22_H_22_O_11_	461.1028 [M − H]^−^	446 [M − H − CH_3_]^−^; 299 [M − H − glc]^−^	463.1251 [M + H]^+^	445; 301
20	27.40	Quercetin-3,7-di-*O*-glucopyranoside	C_27_H_30_O_17_	625.1410 [M − H]^−^	301 [M − H − glc − glc]^−^	627.2453 [M + H]^+^	n.a.
21^a^	27.51	Dihydrokaempferol (compound 8)	C_15_H_12_O_6_	287.0521 [M − H]^−^	269 [M − H − H_2_O]^−^; 243 [M − H − CO_2_]^−^; 161 [M − H − C_6_H_6_O]^−^	289.0719 [M + H]^+^	272
22	27.73	(−)-Epigallocatechin 3-*O*-(*Z*)-*p*-coumaroate (compound 2)	C_24_H_20_O_9_	451.0975 [M − H]^−^	433 [M − H − H_2_O]^−^; 407 [M − H − CO_2_]^−^; 357 [M − H − C_5_H_3_O_2_]^−^; 305 [M − H − C_9_H_6_O_2_]^−^; 287 [M − H − C_9_H_8_O_3_]^-^; 269 [M − H − C_9_H_6_O_4_]^−^; 229; 163 [M − H − C_9_H_8_O_4_] ^−^	453.1199 [M + H]^+^	435; 327; 289; 247; 139
23^a^	28.46	Quercetin (compound 9)	C_15_H_10_O_7_	301.0311 [M − H]^−^	273 [M − H − CO] ^−^; 257 [M − H − OH] ^−^; 151 [M − H − C_8_H_7_O_3_] ^−^	303.0509 [M + H]^+^	n.a.
24	30.15	Kaempferol	C_15_H_10_O_6_	285.0361 [M − H]^−^	257 [M − H − CO]^−^; 241 [M − H − CO_2_]^−^; 151 [M − H − C_8_H_6_O_2_]^−^; 133 [M − H − C_7_H_4_O_4_]^−^;	287.0565 [M + H]^+^	241; 153
25	31.53	Orobol	C_15_H_10_O_6_	285.0364 [M − H]^−^	241 [M − H − CO_2_]^−^; 175 [M − H − B]^−^	287.0558 [M + H]^+^	n.a.
26	32.36	Demethylpraecanson B	C_21_H_20_O_5_	n.a.	n.a.	353.2312 [M + H]^+^	335 [M + H − H_2_O]^+^; 253; 235 [M + H − C_8_H_6_O]^+^;195
27	33.70	Luteolin	C_15_H_10_O_6_	285.0363 [M − H]^−^	241 [M − H − CO_2_]^−^; 175 [M − H − B]^−^; 133 [M − H − C_7_H_4_O_4_]^−^;	287.0561 [M + H]^+^	269; 153; 137
28	34.25	Isoquercitrin	C_21_H_20_O_12_	463.0966 [M − H]^−^	445 [M − H − H_2_O]^−^; 301 [M − H − glc]^−^; 283 [M − H − H_2_O − glc]^−^; 253	465.1192 [M + H]^+^	447; 341; 286; 162
29	36.96	7,2′,4′,5′-Tetramethoxyisoflavon/ 7,2′,3′,4′-Tetramethoxyflavone	C_19_H_18_O_6_	n.a.	n.a.	343.1191 [M + H]^+^	328; 282; 253; 150
30	37.03	Robinetinidol-4alpha-ol	C_15_H_14_O_7_	305.1713 [M − H]^−^	287 [M − H − H_2_O]^−^; 249; 135 [M − H − C_7_H_6_O_5_]^−^	307.2065 [M + H]^+^	n.a.
31	42.53	Norartocarpetin/ 7,8,2′,4′-Tetrahydroxyisoflavone/ 5,7,2′,6′-Tetrahydroxyflavone/ 5,7,2′,3′-Tetrahydroxyflavone/ 5,7,2′,5′-Tetrahydroxyflavone	C_15_H_10_O_6_	285.0360 [M − H]^−^	241 [M − H − CO_2_]^−^; 175 [M − H − B]^−^	n.a.	n.a.
32	47.13	Icariin	C_33_H_40_O_15_	n.a.	n.a.	677.3754 [M + H]^+^	515
33	55.30	Wogonin/Oroxylin A	C_16_H_12_O_5_	283.1661 [M − H]^−^	163; 107	n.a.	n.a.
34	57.52	Nigrolineaxanthone N/Kanzonol M	C_23_H_26_O_6_	397.2534 [M − H]^−^	329	n.a.	n.a.

n.a.: not available.

## Supplementary Materials

The following are available online.
