# Supplementary material for: Phytochemical Composition, Hepatoprotective, and Antioxidant Activities of Phyllodium pulchellum (L.) Desv
Source: Molecules. 2018 Jun 5;23(6):1361. doi: 10.3390/molecules23061361 (PMC6100508; doi:10.3390/molecules23061361)
Supplement: Supplementary file 1 [file molecules-23-01361-s001.pdf]

Supporting Information

Phytochemical and Hepatoprotective and Antioxidant Activities of *Phyllodium pulchellum*

Ya-Chu Fan[a,b], Shi-Jun Yue[a,b], Zhong-Long Guo[a,b], Lan-Ting Xin[a,b], Chao-Yi Wang[a,b], Dong-Lin Zhao[a,c], Hua-Shi Guan[a,b] *, Chang-Yun Wang[a,b] *

[a]*Key Laboratory of Marine Drugs, The Ministry of Education of China, School of Medicine and Pharmacy, Ocean University of China, Qingdao 266003, China*

[b]*Laboratory for Marine Drugs and Bioproducts, Qingdao National Laboratory for Marine Science and Technology, Qingdao 266071, China*

[c]*Marine Agriculture Research Center, Tobacco Research Institute of Chinese Academy of Agricultural Sciences, Qingdao 266101, China*

[*]Corresponding authors. Tel./Fax: +86-532-8203-1536 (C.-Y. Wang); +86-532-8203-1667 (H.-S. Guan).

E-mail address: changyun@ouc.edu.cn (C.-Y. Wang), hsguan@ouc.edu.cn (H.-S. Guan).

List of Supporting Information

NMR and MS data of compounds 1-19

NMR and MS spectra of compounds 1-11 and 16-19

Figure S1. ^{1}H NMR (500 MHz, CD_3OD) spectrum of compound **1**.

Figure S2. ^{13}C NMR (125 MHz, CD_3OD) spectrum of compound **1**.

Figure S3. MS spectrum of compound **1**.

Figure S4. ^{1}H NMR (500 MHz, CD_3OD) spectrum of compound **2**.

Figure S5. ^{13}C NMR (125 MHz, CD_3OD) spectrum of compound **2**.

Figure S6. MS spectrum of compound **2**.

Figure S7. ^{1}H NMR (500 MHz, CD_3OD) spectrum of compound **3**.

Figure S8. ^{13}C NMR (125 MHz, CD_3OD) spectrum of compound **3**.

Figure S9. MS spectrum of compound **3**.

Figure S10. ^{1}H NMR (500 MHz, CD_3OD) spectrum of compound **4**.

Figure S11. ^{13}C NMR (125 MHz, CD_3OD) spectrum of compound **4**.

Figure S12. MS spectrum of compound **4**.

Figure S13. ^{1}H NMR (500 MHz, CD_3OD) spectrum of compound **5**.

Figure S14. ^{13}C NMR (125 MHz, CD_3OD) spectrum of compound **5**.

Figure S15. MS spectrum of compound **5**.

Figure S16. ^{1}H NMR (500 MHz, CD_3OD) spectrum of compound **6**.

Figure S17. ^{13}C NMR (125 MHz, CD_3OD) spectrum of compound **6**.

Figure S18. MS spectrum of compound **6**.

Figure S19. ^{1}H NMR (500 MHz, DMSO-d_6) spectrum of compound **7**.

Figure S20. ^{13}C NMR (500 MHz, DMSO-d_6) spectrum of compound **7**.

Figure S21. MS spectrum of compound **7**.

Figure S22. ^{1}H NMR (500 MHz, DMSO-d_6) spectrum of compound **8**.

Figure S23. ^{13}C NMR (500 MHz, DMSO-d_6) spectrum of compound **8**.

Figure S24. MS spectrum of compound **8**.

Figure S25. ^{1}H NMR (500 MHz, CD_3OD) spectrum of compound **9**.

Figure S26. ^{13}C NMR (500 MHz, CD_3OD) spectrum of compound **9**.

Figure S27. MS spectrum of compound **9**.

Figure S28. ^{1}H NMR (500 MHz, DMSO-d_6) spectrum of compound **10**.

Figure S29. ^{13}C NMR (500 MHz, DMSO-d_6) spectrum of compound **10**.

Figure S30. MS spectrum of compound **10**.

Figure S31. ^{1}H NMR (500 MHz, DMSO-d_6) spectrum of compound **11**.

Figure S32. ^{13}C NMR (500 MHz, DMSO-d_6) spectrum of compound **11**.

Figure S33. MS spectrum of compound **11**.

Figure S34. ^{1}H NMR (500 MHz, DMSO-d_6) spectrum of compound **16**.

Figure S35. ^{13}C NMR (500 MHz, DMSO-d_6) spectrum of compound **16**.

Figure S36. MS spectrum of compound **16**.

Figure S37. ^{1}H NMR (500 MHz, DMSO-d_6) spectrum of compound **17**.

Figure S38. ^{13}C NMR (500 MHz, DMSO-d_6) spectrum of compound **17**.

Figure S39. MS spectrum of compound **17**.

Figure S40. ^{1}H NMR (500 MHz, DMSO-d_6) spectrum of compound **18**.

Figure S41. ^{13}C NMR (500 MHz, DMSO-d_6) spectrum of compound **18**.

Figure S42. MS spectrum of compound **18**.

Figure S43. ^{1}H NMR (500 MHz, DMSO-d_6) spectrum of compound **19**.

Figure S44. ^{13}C NMR (500 MHz, DMSO-d_6) spectrum of compound **19**.

Figure S45. MS spectrum of compound **19**.

The possible fragmentation pathway for compound 2

Figure S46. The possible fragmentation pathway for compound **2**.

NMR and MS data of compounds 1-19

(–)-epigallocatechin 3-*O*-(*E*)-*p*-coumaroate (1): brown amorphous powder; $[\alpha]^{22}_{D}$: –204.5 (*c* 0.25, MeOH). ^{1}H NMR (500 MHz, CD_3OD, *δ* ppm): 7.47 (1H, d, *J* = 15.9 Hz, H-7″), 7.38 (2H, d, *J* = 8.4 Hz, H-2″, H-6″), 6.75 (2H, d, *J* = 8.4 Hz, H-3″, H-5″), 6.51 (2H, s, H-2′, H-6′), 6.23 (1H, d, *J* = 15.9 Hz, H-8″), 5.97 (1H, d, *J* = 2.2 Hz, H-6), 5.95 (1H, d, *J* = 2.2 Hz, H-8), 5.45 (1H, m, H-3), 4.93(1H, br s, H-2), 2.96 (1H, dd, *J* = 17.3, 4.6 Hz, H-4a), 2.84 (1H, dd, *J* = 17.3, 2.0 Hz, H-4b). ^{13}C NMR (125 MHz, CD_3OD): 168.6 (C, C-9″), 161.1 (C, C-4″), 157.8 (C, C-5), 157.8 (C, C-7), 157.1 (C, C-9), 146.8 (C, C-4′), 146.7 (C, C-3′), 146.7 (C, C-5′), 133.7 (CH, C-7″), 131.2 (CH, C-2″), 131.2 (CH, C-6″), 130.7 (C, C-1′), 127.2 (C, C-1″), 116.7 (CH, C-2′), 116.7 (CH, C-6′), 115.1 (CH, C-8″), 106.8 (CH, C-3″), 106.8 (CH, C-5″), 99.4 (C, C-9), 96.5 (CH, C-6), 95.8 (CH, C-8), 78.4 (CH, C-2), 69.8 (CH, C-3), 26.7 (CH_2, C-4). ESIMS *m/z* 453.1 $[M + H]^+$, 475.1 $[M + Na]^+$.

(–)-epigallocatechin 3-*O*-(*Z*)-*p*-coumaroate (2): brown amorphous powder; $[\alpha]^{22}_{D}$: –190.5 (*c* 0.25, MeOH). ^{1}H NMR (500 MHz, CD_3OD, *δ* ppm): 7.36 (2H, d, *J* = 8.6 Hz, H-2″, 6″), 6.75 (1H, d, *J* = 12.7 Hz, H-7″), 6.65 (2H, d, *J* = 8.6 Hz, H-3″, H-5″), 6.49 (2H, s, H-2′, H-6′), 5.95 (1H, d, *J* = 2.2 Hz, H-6), 5.92 (1H, d, *J*= 2.2 Hz, H-8), 5.66 (1H, d, *J* = 12.7Hz, H-8″), 5.46 (1H, m, H-3), 4.92(1H, br s, H-2), 2.95 (1H, dd, *J* = 17.5, 4.7 Hz, H-4a), 2.84 (1H, dd, *J* = 17.5, 2.0 Hz, H-4b). ^{13}C NMR (125 MHz, CD_3OD): 167.6 (C, C-9″), 159.8 (C, C-4″), 157.9 (C, C-5), 157.8 (C, C-7), 157.1 (C, C-9), 146.7 (C, C-3′), 146.7 (C, C-5′), 144.8 (C, C-4′), 133.8 (CH, C-7″), 133.4 (CH, C-2″), 133.4 (CH, C-6″), 130.7 (C, C-1′), 127.5 (C, C-1″), 116.6 (CH, C-8″), 115.8 (CH, C-2′), 115.8 (CH, C-6′), 106.8 (CH, C-3″), 106.8 (CH, C-5″), 99.3 (C, C-10), 96.5 (CH, C-6), 95.9 (CH, C-8), 78.4 (CH, C-2), 69.5 (CH, C-3), 26.7 (CH_2, C-4). ESIMS *m/z* 453.1 $[M + H]^+$, 475.1 $[M + Na]^+$.

(–)-gallocatechin (3): white powder; $[\alpha]^{22}_{D}$: –16.5 (*c* 0.1, MeOH). ^{1}H NMR (500 MHz, CD_3OD, *δ* ppm): 6.39 (2H, s, H-2′, H-6′), 5.91 (1H, s, H-8), 5.85 (1H, s, H-6), 4.52 (1H, d, *J* = 7.5 Hz, H-2), 3.95 (1H, m, H-3), 2.80 (1H, dd, *J* = 16.1, 5.1 Hz, H-4a), 2.49 (1H, dd, *J* = 16.1, 8.0Hz, H-4b). ^{13}C NMR (125 MHz, CD_3OD): 157.8 (C, C-7), 157.6 (C, C-5), 156.8 (C, C-9), 146.8 (C, C-3′), 146.8 (C, C-5′), 134.0 (C, C-4′),

131.6 (C, C-1′), 107.2 (CH, C-2′), 107.2 (CH, C-6′), 100.7 (C, C-10), 96.3 (CH, C-8), 95.5 (CH, C-6), 82.9 (CH, C-2), 68.8 (CH, C-3), 28.1 (CH_2, C-4). ESI-MS: *m/z* 307.1 $[M + H]^+$, 345.0 $[M + K]^+$.

(+)-catechin (4): white powder; $[\alpha]^{22}_D$: +0.9 (*c* 0.1, MeOH). ^1H NMR (500 MHz, CD_3OD, *δ* ppm): 6.83 (1H, d, *J* = 1.5 Hz, H-2′), 6.75 (1H, d, *J* = 8.2 Hz, H-5′), 6.71 (1H, dd, *J* = 8.2, 1.5 Hz, H-6′), 5.92 (1H, d, *J* = 2.0 Hz, H-8), 5.84 (1H, d, *J* = 2.0 Hz, H-6), 4.55 (1H, s, H-2), 3.96 (1H, m, H-3), 2.84 (1H, dd, *J* = 16.1, 5.5 Hz, H-4a), 2.49 (1H, dd, *J* = 16.1, 8.1 Hz, H-4b). ^{13}C NMR (125 MHz, CD_3OD): 157.8 (C, C-7), 157.6 (C, C-5), 156.9 (C, C-9), 146.2 (C, C-3′), 146.2 (C, C-4′), 132.2 (C, C-1′), 120.2 (CH, C-6′), 116.1 (CH, C-2′), 115.2 (C, C-5′), 100.8 (C, C-10), 96.3 (CH, C-8), 95.5 (CH, C-6), 82.8 (CH, C-2), 68.8 (CH, C-3), 28.5 (CH_2, C-4). ESI-MS: *m/z* 291.1 $[M + H]^+$, 313.1 $[M + Na]^+$.

(–)-epigallocatechin (5): white powder; $[\alpha]^{22}_D$: –47.2 (*c* 0.1, MeOH). ^1H NMR (500 MHz, CD_3OD, *δ* ppm): 6.51 (2H, s, H-2′, H-6′), 5.93 (1H, s, H-8), 5.91 (1H, s, H-6), 4.75 (1H, s, H-2), 4.16 (1H, m, H-3), 2.84 (1H, dd, *J* = 16.7, 4.5 Hz, H-4a), 2.72 (1H, dd, *J* = 16.7, 2.14 Hz, H-4b). ^{13}C NMR (125 MHz, CD_3OD): 158.0 (C, C-7), 157.7 (C, C-5), 157.3 (C, C-9), 146.7 (C, C-3′), 146.7 (C, C-5′), 133.6 (C, C-4′), 131.5 (C, C-1′), 107.0 (CH, C-2′), 107.0 (CH, C-6′), 100.1 (C, C-10), 96.4 (CH, C-8), 95.9 (CH, C-6), 79.9 (CH, C-2), 67.5 (CH, C-3), 29.1 (CH_2, C-4). ESI-MS: *m/z* 307.1 $[M + H]^+$, 329.0 $[M + Na]^+$.

(–)-epicatechin (6): white powder; $[\alpha]^{22}_D$: –70.1 (*c* 0.1, MeOH). ^1H NMR (500 MHz, CD_3OD, *δ* ppm): 6.97 (1H, d, *J* = 1.2 Hz, H-2′), 6.79 (1H, d, *J* = 8.2 Hz, H-5′), 6.75 (1H, dd, *J* = 8.2, 1.2 Hz, H-6′), 5.94 (1H, d, *J* = 2.0 Hz, H-8), 5.92 (1H, d, *J* = 2.0 Hz, H-6), 4.80 (1H, s, H-2), 4.16 (1H, m, H-3), 2.85 (1H, dd, *J* = 16.7, 4.4 Hz, H-4a), 2.73 (1H, dd, *J* = 16.7, 2.0 Hz, H-4b). ^{13}C NMR (125 MHz, CD_3OD): 157.9 (C, C-7), 157.5 (C, C-5), 157.3 (C, C-9), 145.8 (C, C-3′), 145.7 (C, C-4′), 132.2 (C, C-1′), 119.4 (CH, C-6′), 115.9 (CH, C-2′), 115.3 (C, C-5′), 100.1 (C, C-10), 96.4 (CH, C-8), 95.9 (CH, C-6), 79.8 (CH, C-2), 67.4 (CH, C-3), 29.3 (CH_2, C-4). ESI-MS: *m/z* 291.1 $[M + H]^+$, 581.1 $[2M + H]^+$.

Dihydroquercetin (7): yellow powder; $[\alpha]^{22}_D$: +25.6, ^1H NMR (500 MHz, DMSO-d_6,

δ ppm): 11.89 (1H, s, 5-OH), 10.96 (1H, s, 7-OH), 9.05, 8.81 (2H, s, 3′-OH, 4′-OH), 6.88 (1H, s, H-2′), 6.74 (2H, br s, H-5′, H-6′), 5.94 (1H, d, J = 1.8 Hz, H-8), 5.89 (1H, d, J = 1.8 Hz, H-6), 4.97 (1H, dd, J = 11.1 Hz, H-2), 4.49 (1H, d, J = 11.1 Hz, H-3). ^{13}C NMR (125 MHz, DMSO-d_6): 197.7 (C, C-4), 166.9 (C, C-7), 163.3 (C, C-5), 162.5 (C, C-9), 145.8 (C, C-4′), 144.9 (C, C-3′), 128.0 (CH, C-1′), 119.3 (CH, C-6′), 115.4 (CH, C-2′), 115.2 (CH, C-5′), 100.4 (C, C-10), 96.0 (CH, C-6), 95.0 (CH, C-8), 83.0 (CH, C-2), 71.6 (CH, C-3). HRESI-MS m/z 303.0465 [M − H]$^-$ (calcd for $C_{15}H_{11}O_7$, 303.0499).

(+)-dihydrokaempferol (8): yellow powder; $[\alpha]^{22}_{D}$: +20.2 (c 1.0, MeOH). ^{1}H NMR (500 MHz, DMSO-d_6, δ ppm): 7.28 (2H, d, J = 8.5, 2.0 Hz, H-2′, H-6′), 6.76 (2H, d, J = 8.5, 2.0 Hz, H-3′, H-5′), 5.55 (1H, d, J = 1.2 Hz, H-8), 5.51 (1H, d, J = 1.2 Hz, H-6), 4.89 (1H, dd, J = 11.4 Hz, H-2), 4.39 (1H, d, J = 11.4 Hz, H-3).^{13}C NMR (125 MHz, DMSO-d_6): 197.9 (C, C-4), 166.6(C, C-7), 163.4 (C, C-5), 162.1 (C, C-9), 157.6 (C, C-4′), 129.3 (CH, C-6′), 129.3 (CH, C-2′), 128.2 (C, C-1′), 114.8 (C, C-3′), 114.8 (C, C-5′), 100.7 (C, C-10), 97.6 (CH, C-6), 96.9 (CH, C-8), 82.4 (CH, C-2), 71.2 (CH, C-3). HRESI-MS m/z 287.0521 [M − H]$^-$ (calcd for $C_{15}H_{11}O_6$, 287.0561).

Quercetin (9): yellow powder; ^{1}H NMR (500 MHz, CD_3OD, δ ppm): 7.73 (1H, d, J = 2.0 Hz, H-2′), 7.62 (1H, dd, J = 2.0, 8.5 Hz, H-6′), 6.88 (1H, d, J = 8.5 Hz, H-5′), 6.38 (1H, d, J = 1.8 Hz, H-8), 6.17 (1H, d, J = 1.8 Hz, H-6) ^{13}C NMR (125 MHz, CD_3OD): 177.3 (C, C-4), 165.5 (C, C-7), 162.4 (C, C-9), 158.2 (C, C-5), 148.7 (C, C-4′), 148.0 (C, C-2), 146.2 (C, C-3′), 137.2 (C, C-3), 124.1 (C, C-1′), 121.7 (CH, C-6′), 116.2 (CH, C-5′), 116.0 (CH, C-2′), 104.5 (C, C-10), 99.2 (CH, C-6), 94.4 (CH, C-8). HRESI-MS m/z 303.0513 [M + H]$^+$ (calcd for $C_{15}H_{11}O_7$, 303.0499).

Rutin (10): yellow powder; ^{1}H NMR (500 MHz, DMSO-d_6, δ ppm): 12.60 (1H, s, 5-OH), 7.55 (1H, d, J = 2.0 Hz, H-2′), 7.53 (1H, dd, J = 2.0, 8.4 Hz, H-6′), 6.84 (1H, d, J = 8.4 Hz, H-5′), 6.38 (1H, d, J = 2.0 Hz, H-8), 6.19 (1H, d, J = 2.0 Hz, H-6), 5.35 (1H, d, J = 7.4 Hz, H-1″), 4.38 (1H, d, J = 1.2 Hz, H-1‴), 0.99 (3H, d, J = 6. 2 Hz, -CH_3). ^{13}C NMR (125 MHz, DMSO-d_6): 177.3 (C, C-4), 164.0 (C, C-7), 161.2 (C, C-5), 156.6 (C, C-2), 156.4 (C, C-9), 148.4 (C, C-4′), 144.7 (C, C-3′), 133.3 (C, C-3), 121.5 (CH, C-6′), 121.1 (C, C-1′), 116.2 (CH, C-5′), 115.2 (CH, C-2′), 103.9 (C,

C-10), 101.1 (CH, C-1″), 100.7 (CH, C-1‴), 98.6 (CH, C-6), 93.5 (CH, C-8), 76.4 (CH, C-3″), 75.9 (CH, C-5″), 74.0 (CH, C-2″), 71.8 (CH, C-4‴), 70.5 (CH, C-2‴), 70.3 (CH, C-3‴), 70.0 (CH, C-4″), 68.2 (CH, C-5‴), 67.0 (CH, C-6″), 17.7 (CH, C-6‴). ESI-MS: *m/z* 611.0 $[M + H]^+$, 633.0 $[M + Na]^+$.

Duercetin-3-O-*α*-*L*-rhamnopyranosyl-(1→6)-*β*-*D*-galactopyranoside (11): yellow powder; ^{1}H NMR (500 MHz, DMSO-d_6, δ ppm): 7.62 (1H, dd, J = 2.0, 8.5 Hz, H-6′), 7.49 (1H, d, J = 2.0 Hz, H-2′), 6.76 (1H, d, J = 8.5 Hz, H-5′), 6.19 (1H, d, J = 2.0 Hz, H-8), 6.01 (1H, d, J = 2.0 Hz, H-6), 5.21 (1H, d, J = 7.7 Hz, H-1″), 4.42 (1H, s, H-1‴), 0.91 (3H, d, J = 6.8 Hz, H-6‴). ^{13}C NMR (125 MHz, DMSO-d_6): 176.4 (C, C-4), 160.9 (C, C-7), 160.9 (C, C-5), 156.7 (C, C-2), 156.7 (C, C-9), 145.1 (C, C-4′), 145.1 (C, C-3′), 133.2 (C, C-3), 121.9 (CH, C-6′), 121.9 (C, C-1′), 115.4 (CH, C-5′), 115.1 (CH, C-2′), 102.8 (C, C-10), 100.1 (CH, C-1″), 100.1 (CH, C-1‴), 94.2 (CH, C-8), 73.3 (CH, C-5″), 73.1 (CH, C-3″), 71.9 (CH, C-4‴), 71.1 (CH, C-2″), 70.6 (CH, C-2‴), 70.4 (CH, C-3‴), 68.2 (CH, C-5‴), 68.0 (CH, C-4″), 65.2 (CH, C-6″), 17.9 (CH, C-6‴). HRESI-MS *m/z* 611.1634 $[M + H]^+$ (calcd for $C_{27}H_{31}O_{16}$, 611.1607).

5-Hydroxy-*N*,*N*-dimethyltryptamine (12): colorless crystal; ^{1}H NMR (500 MHz, DMSO-d_6, δ ppm): 9.82 (1H, s, H-1), 6.44 (1H, d, J = 8.6 Hz, H-7), 6.33 (1H, s, H-2), 6.15 (1H, d, J = 2.0 Hz, H-4), 5.93 (1H, dd, J = 8.6, 2.0 Hz, H-6), 2.05 (2H, m, H-11), 1.82 (2H, m, H-10), 1.54 (6H, s, H-13, H-14). ^{13}C NMR (125 MHz, DMSO-d_6): 150.2 (C, C-5), 130.9 (C, C-8), 128.0 (C, C-9), 123.0 (CH, C-2), 111.7 (CH, C-7), 111.5 (CH, C-3), 111.3 (C, C-6), 102.3 (CH, C-4), 60.0 (CH_2, C-11), 45.1 (CH_3, C-13), 45.1 (CH_3, C-14), 23.2 (CH_2, C-10). ESI-MS: *m/z* 205.2 $[M + H]^+$.

5-Methoxy-*N*,*N*-dimethyltryptamine (13): colorless crystal; ^{1}H NMR (500 MHz, DMSO-d_6, δ ppm): 10.73 (1H, s, H-1), 7.34 (1H, d, J = 8.6 Hz,, H-7), 7.20 (1H, s, H-2), 7.09 (1H, d, J = 2.0 Hz, H-4), 6.83 (1H, dd, J = 8.6, 2.0 Hz, H-6), 3.87 (3H, s, CH_3O-5), 2.90 (2H, m, H-11), 2.63 (2H, m, H-10), 2.34 (6H, s, H-13, H-14). ^{13}C NMR (125 MHz, DMSO-d_6): 155.2 (C, C-5), 133.3 (C, C-8), 128.7 (C, C-9), 124.1 (CH, C-2), 112.9 (CH, C-7), 114.0 (CH, C-3), 112.9 (C, C-6), 101.3 (CH, C-4), 60.9 (CH_2, C-11), 55.6 (CH_3, CH_3O-5), 44.5 (CH_3, C-13), 44.5 (CH_3, C-14), 18.9 (CH_2, C-10). ESI-MS: *m/z* 219.2 $[M + H]^+$.

5-Hydroxy-*N,N*-dimethyltryptamine-oxide (14): colorless crystal; ^{1}H NMR (500 MHz, DMSO-d_6, δ ppm): 10.66 (1H, s, H-1), 7.14 (1H, d, J = 8.6 Hz,, H-7), 7.10 (1H, s, H-2), 6.92 (1H, d, J = 2.0 Hz, H-4), 6.63 (1H, dd, J = 8.6, 2.0 Hz, H-6), 3.60 (2H, d, J = 7.6 Hz, H-11), 3.34 (6H, s, H-13, H-14), 3.13 (2H, d, J = 7.6 Hz, H-10). ^{13}C NMR (125 MHz, DMSO-d_6): 151.0 (C, C-5), 132.9 (C, C-8), 128.7 (C, C-9), 123.1 (CH, C-2), 112.9 (CH, C-7), 112.0 (CH, C-3), 112.7 (C, C-6), 103.3 (CH, C-4), 69.1 (CH_2, C-11), 57.0 (CH_3, C-13), 57.0 (CH_3, C-14), 20.5 (CH_2, C-10). ESI-MS: *m/z* 221.2 [M + H]$^+$.

5-Methoxy-*N,N*-dimethyltryptamine-oxide (15): colorless crystal; ^{1}H NMR (500 MHz, CD_3OD-d_6, δ ppm): 7.17 (1H, d, J = 8.6 Hz, H-7), 7.08 (1H, s, H-2), 7.06 (1H, d, J = 2.0 Hz, H-4), 6.72 (1H, dd, J = 8.6, 2.0 Hz, H-6), 3.78 (3H, s, CH_3O-5), 3.51 (2H, m, H-11), 3.25 (2H, m, H-10), 3.19 (6H, s, H-13, H-14). ESI-MS: *m/z* 235.2 [M + H]$^+$.

L-tryptophan (16): pale yellow powder; $[\alpha]^{22}_D$: –32 (*c* 10.1, MeOH). ^{1}H NMR (500 MHz, DMSO-d_6, δ ppm): 10.91 (1H, s, H-1), 7.56 (1H, d, J = 8.0 Hz, H-4), 7.35 (1H, d, J = 8.0 Hz,, H-7), 7.20 (1H, s, H-2), 7.06 (1H, t, J = 7.5 Hz, H-6), 6.98 (1H, t, J = 7.5 Hz, H-5), 3.46 (1H, dd, J = 4.8, 8.5 Hz, H-11), 3.31 (1H, dd, J = 4.8, 15.0 Hz, H-10a), 2.97 (1H, dd, J = 8.5, 15.0 Hz, H-10b); ^{13}C NMR (125 MHz, DMSO-d_6): 136.3 (C, C-8), 127.2 (C, C-9), 124.0 (CH, C-2), 120.9 (CH, C-6), 118.4 (CH, C-4), 118.3 (CH, C-5), 111.3 (CH, C-7), 109.6 (C, C-3), 54.7 (CH, C-11), 27.1 (CH_2, C-10), ESI-MS: *m/z* 205.2 [M + H]$^+$.

***N,N*-dimethyl-*L*-tryptophan (17)**: pale yellow powder; ^{1}H NMR (500 MHz, DMSO-d_6, δ ppm): 10.80 (1H, s, H-1), 7.53 (1H, d, J = 8.0 Hz, H-4), 7.32 (1H, d, J = 8.0 Hz, H-7), 7.15 (1H, s, H-2), 7.04 (1H, t, J = 7.5 Hz, H-6), 6.96 (1H, t, J = 7.5 Hz, H-5), 3.41 (1H, dd, J = 4.8, 8.5 Hz, H-11), 3.13 (1H, dd, J = 4.8, 15.0 Hz, H-10a), 2.95 (1H, dd, J = 8.5, 15.0 Hz, H-10b), 2.39 (6H, s, H-13, H-14); ^{13}C NMR (125 MHz, DMSO-d_6): 136.0 (C, C-8), 127.2 (C, C-9), 123.4 (CH, C-2), 120.8 (CH, C-6), 118.2 (CH, C-4), 118.2 (CH, C-5), 111.3 (CH, C-7), 110.8 (C, C-3), 68.8 (CH, C-11), 41.4 (C, C-13, 14), 24.4 (CH_2, C-10), ESI-MS: *m/z* 233.2 [M + H]$^+$.

2-(Indol-3-yl)ethyl-*α-L*-rhamnopyranosyl-(1→6)-*β-D*-glucopyranoside (18) light

brown gummy material; $[\alpha]^{22}_{D}$: −50.4 (*c* 0.1, MeOH), ^{1}H NMR (500 MHz, DMSO-d_6, *δ* ppm): 7.50 (1H, d, *J* = 8.0 Hz, H-4), 7.32 (1H, d, *J* = 8.0 Hz, H-7), 7.18 (1H, s, H-2), 7.05 (1H, t, *J* = 7.5 Hz, H-6), 6.98 (1H, t, *J* = 7.5 Hz, H-5), 4.59 (1H, br s, H-1″), 4.22 (1H, d, *J* = 7.8 Hz, H-1′), 3.94 (1H, dd, *J* = 16.8, 7.5 Hz, H-11a), 3.79 (1H, dd, *J* = 10.8 Hz, H-6′a), 3.70 (1H, dd, *J* = 16.8, 7.5 Hz, H-11b), 2.96-3.61 (m, sugar), 3.16 (1H, m, H-10a), 2.93 (1H, d, *J* = 7.5 Hz, H-10b), 1.11 (3H, d, *J* = 6.2 Hz, H-6″). ^{13}C NMR (125 MHz, DMSO-d_6): 136.3 (C, C-8), 127.6 (C, C-9), 123.2 (CH, C-2), 121.4 (CH, C-6), 118.8 (CH, C-4), 118.7 (CH, C-5), 111.7 (CH, C-7), 111.3 (C, C-3), 103.2 (CH, C-1′), 101.5 (CH, C-1″), 76.8 (CH, C-3′), 75.6 (CH, C-5′), 73.7 (CH, C-2′), 72.2 (CH, C-4″), 70.8 (CH, C-2″), 70.7 (CH, C-3″), 70.4 (CH, C-4′), 69.6 (CH_2, C-11), 68.6 (CH, C-5″), 67.3 (CH_2, C-6′), 25.8 (CH_2, C-10), 18.2 (CH_3, C-6″). HRESI-MS *m/z* 470.2010 $[M + H]^+$ (calcd for $C_{22}H_{32}O_{10}N$, 470.2021).

2-(Indol-3-yl)ethyl-*β-D*-glucopyranoside (**19**): light brown gummy material; $[\alpha]^{22}_{D}$: −32.7 (*c* 0.1, MeOH), ^{1}H NMR (500 MHz, DMSO-d_6, *δ* ppm): 10.80 (1H, s, H-1), 7.52 (1H, d, *J* = 8.0 Hz, H-4), 7.33 (1H, d, *J* = 8.0 Hz,, H-7), 7.20 (1H, s, H-2), 7.05 (1H, t, *J* = 7.5 Hz, H-6), 6.97 (1H, t, *J* = 7.5 Hz, H-5), 4.23 (1H, d, *J* = 7.8 Hz, H-1′), 2.96-4.01 (m, sugar, H-10, H-11); ^{13}C NMR (125 MHz, DMSO-d_6): 136.1 (C, C-8), 127.3 (C, C-9), 123.1 (CH, C-2), 120.9 (CH, C-6), 118.3 (CH, C-4), 118.3 (CH, C-5), 111.4 (CH, C-7), 110.9 (C, C-3), 103.0 (CH, C-1′), 77.0 (CH, C-5′), 76.9 (CH, C-3′), 73.6 (CH, C-2′), 70.2 (CH, C-4′), 69.2 (CH_2, C-11), 61.2 (CH_2, C-6′), 25.5 (CH_2, C-10). ESI-MS: *m/z* 324.3 $[M + H]^+$, 346.2 $[M + Na]^+$.

NMR and MS spectra of compounds 1-11 and 16-19

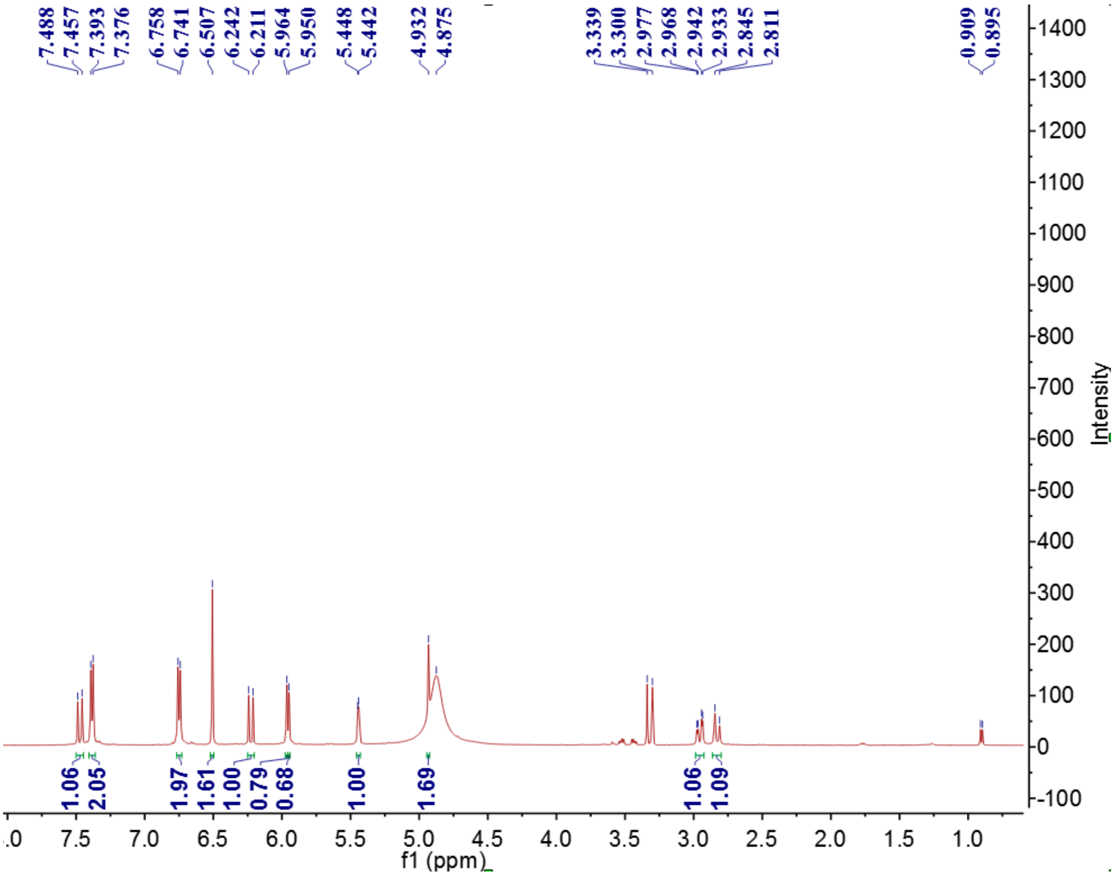

Figure S1. ^{1}H NMR (500 MHz, CD_3OD) spectrum of compound **1**.

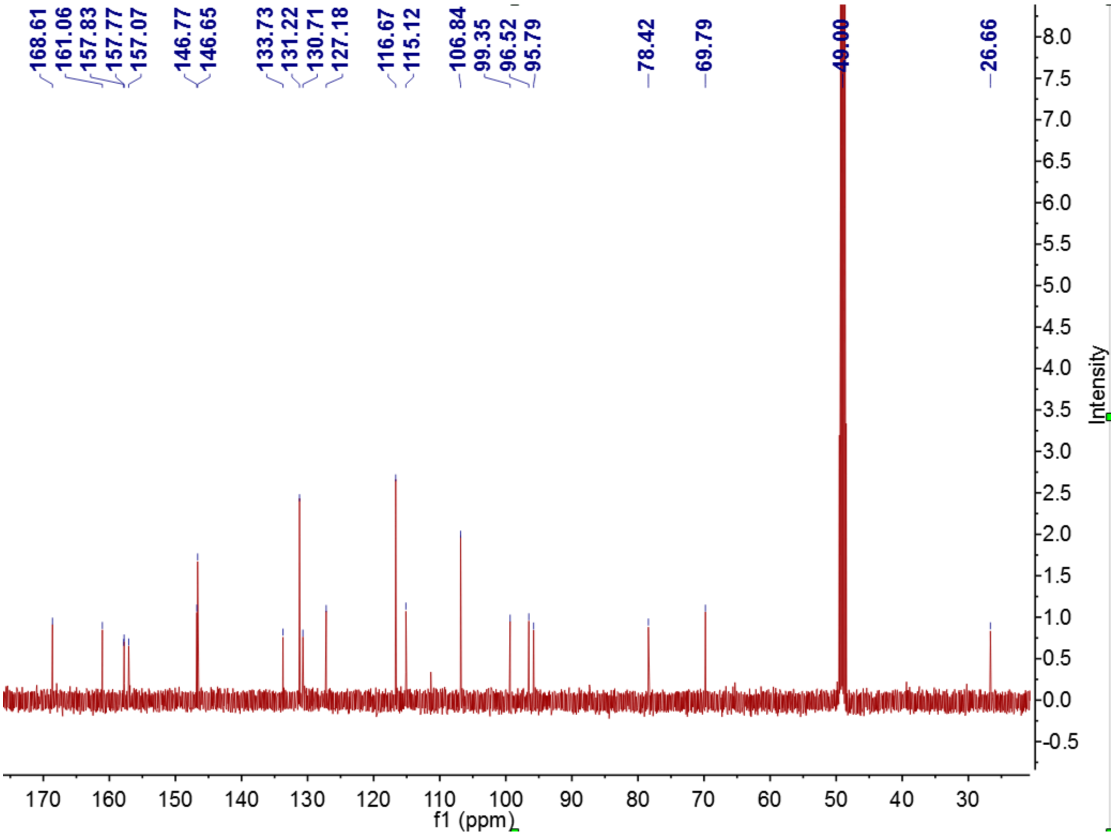

Figure S2. ^{13}C NMR (125 MHz, CD$_3$OD) spectrum of compound **1**.

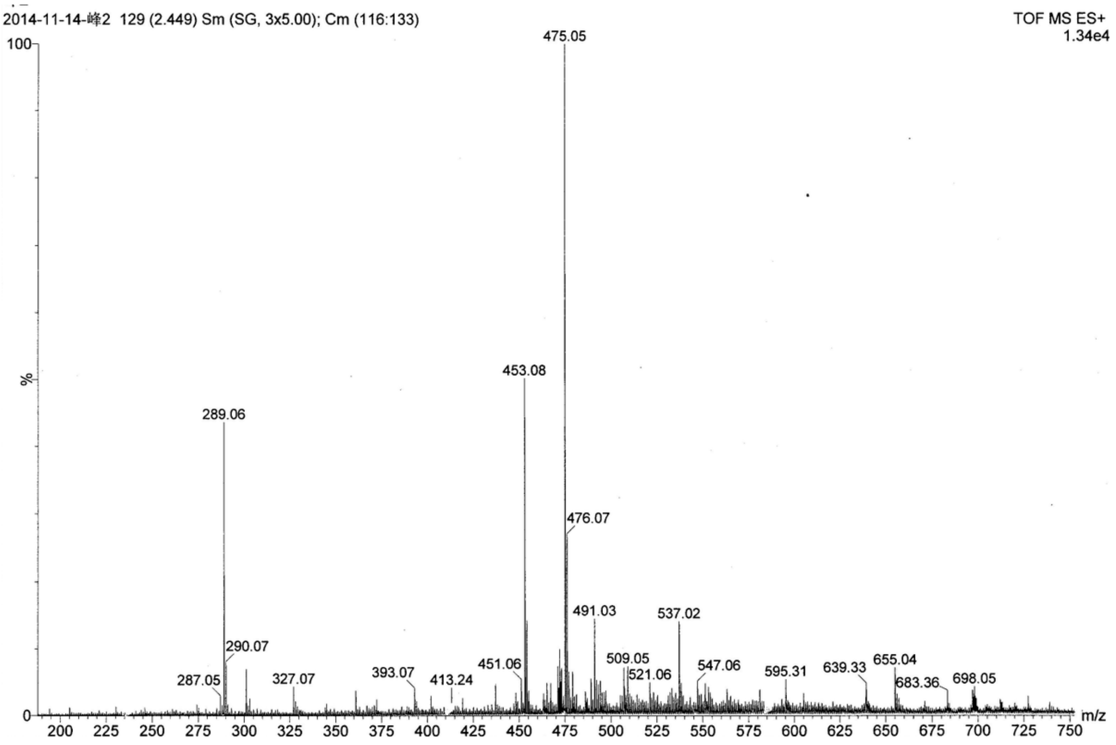

Figure S3. MS spectrum of compound **1**.

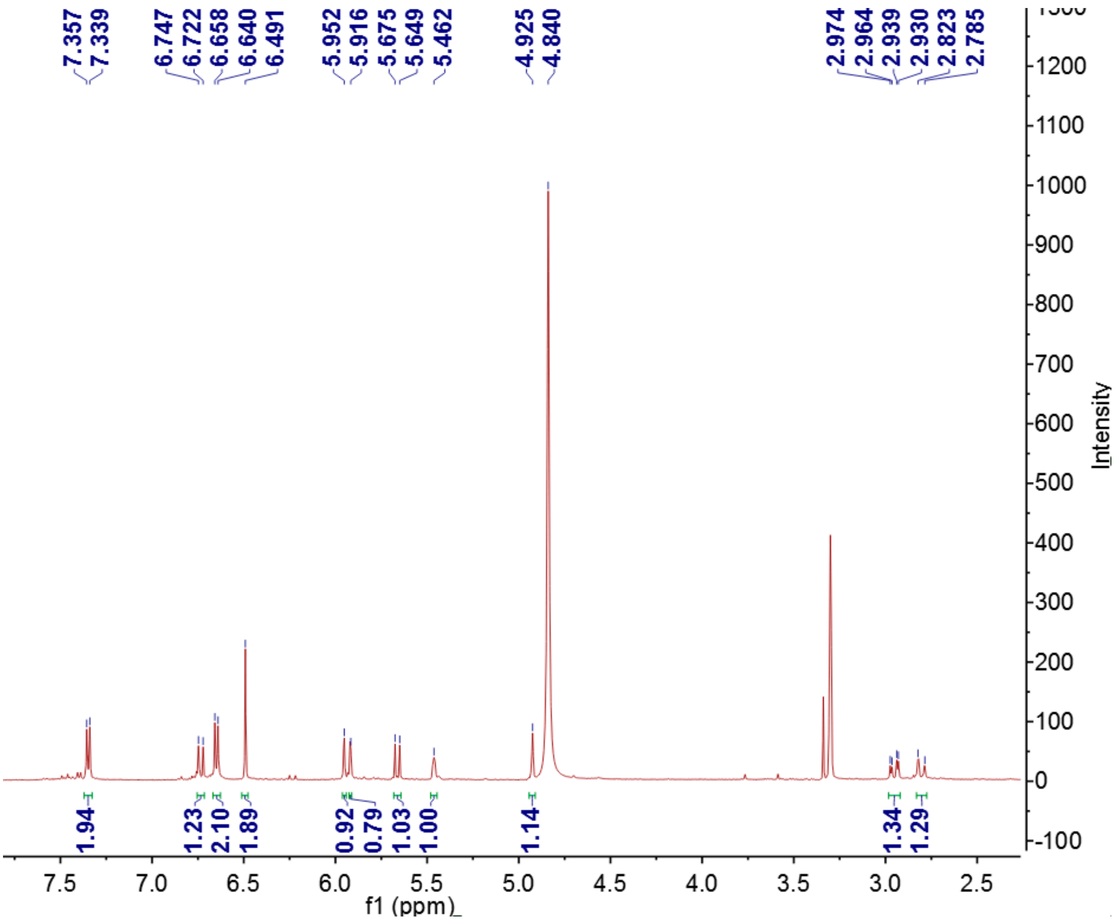

Figure S4. ^{1}H NMR (500 MHz, CD$_3$OD) spectrum of compound **2**.

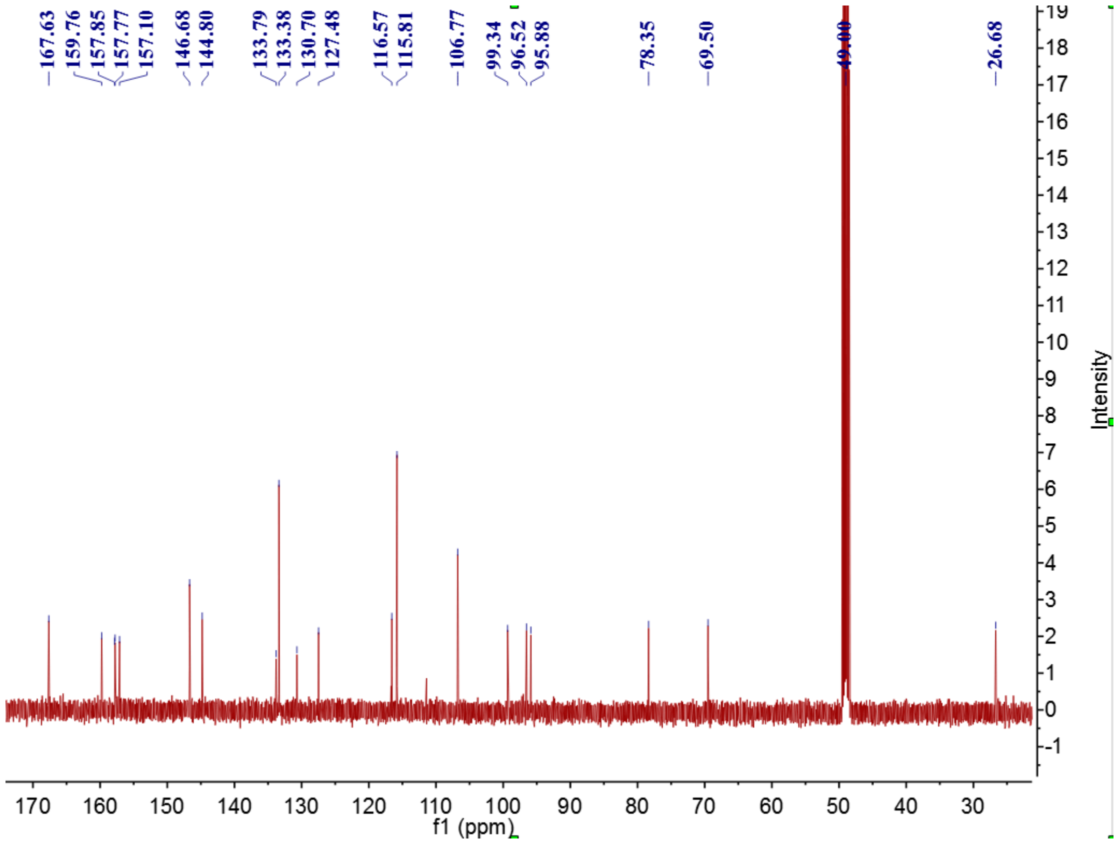

Figure S5. ^{13}C NMR (125 MHz, CD_3OD) spectrum of compound **2**.

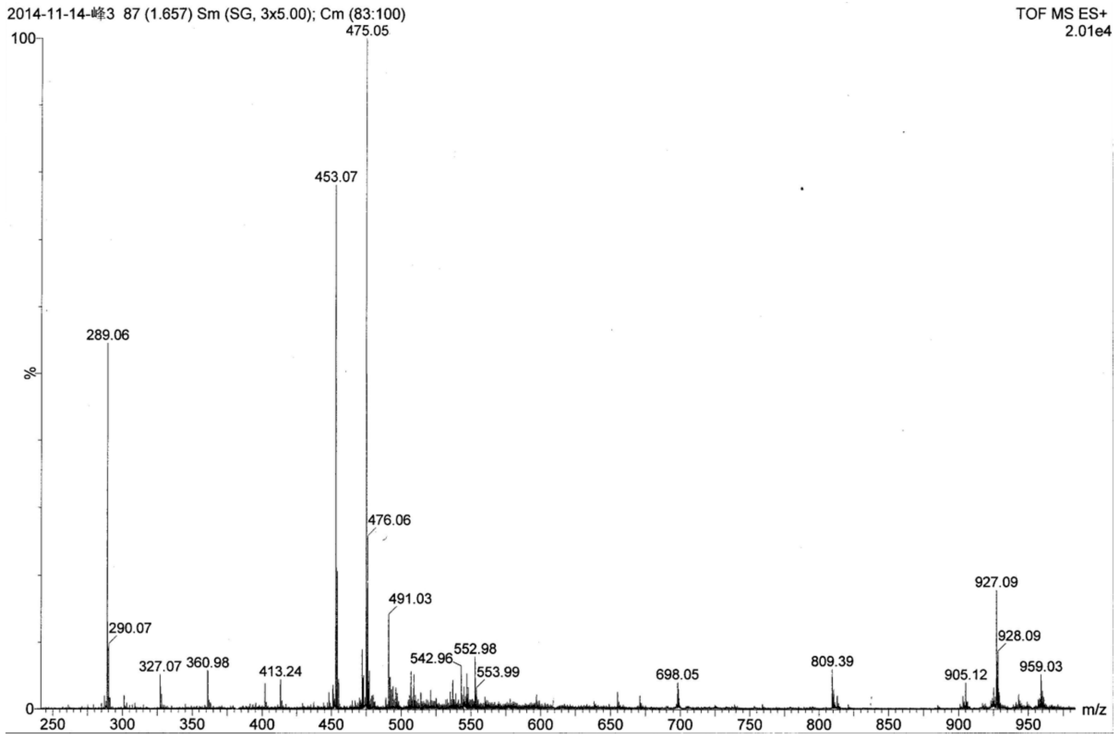

Figure S6. MS spectrum of compound **2**.

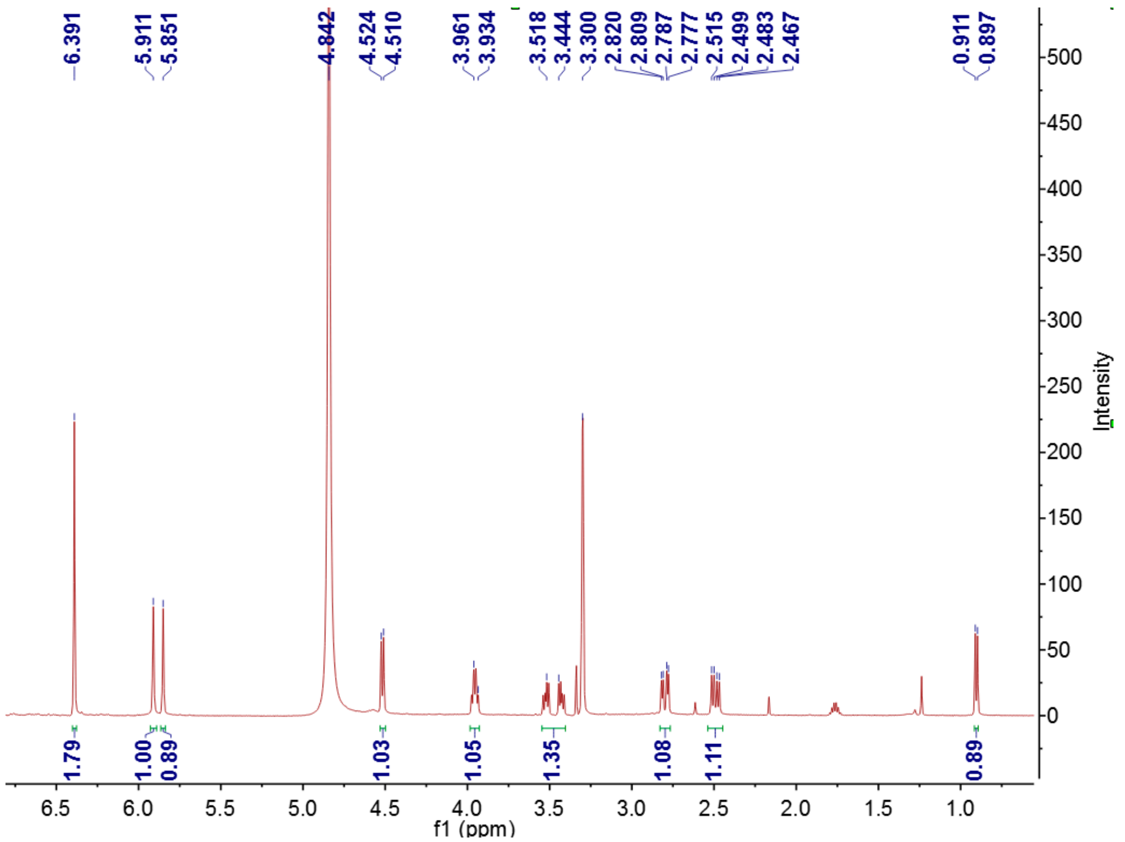

Figure S7. ^{1}H NMR (500 MHz, CD_3OD) spectrum of compound **3**.

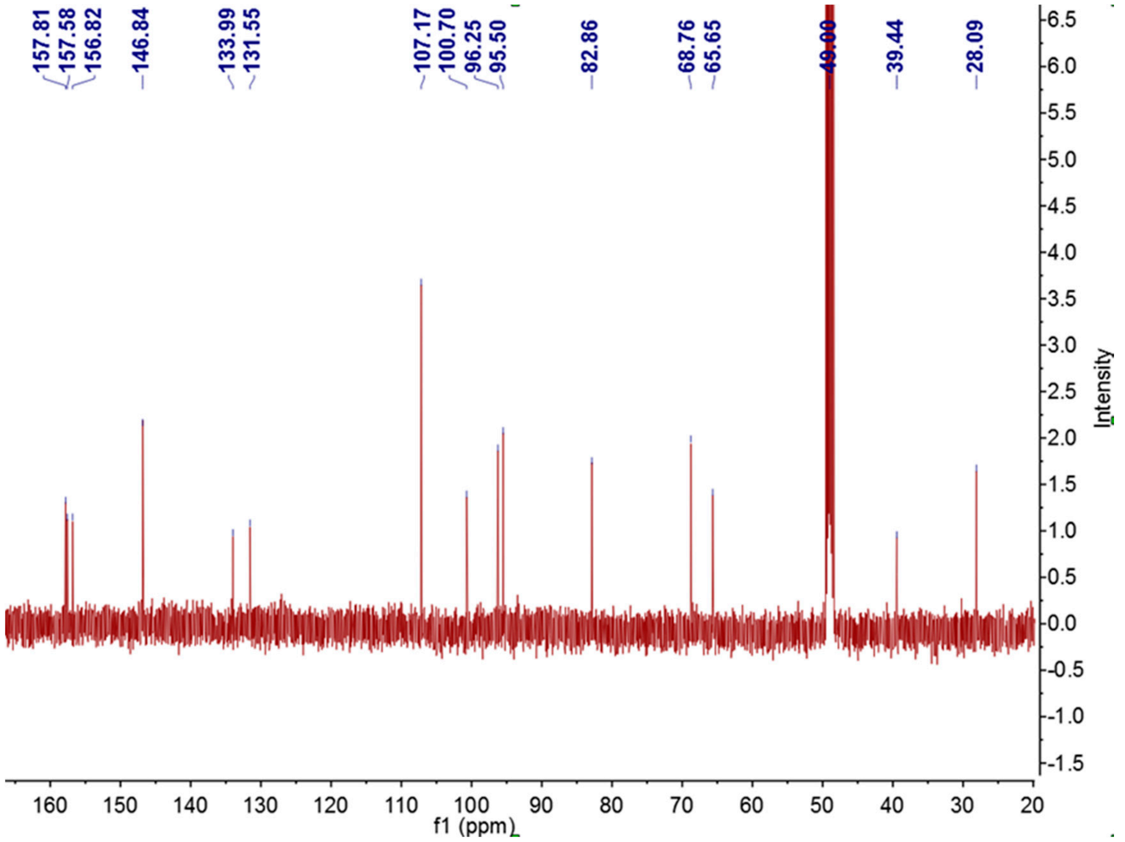

Figure S8. ^{13}C NMR (125 MHz, CD_3OD) spectrum of compound **3**.

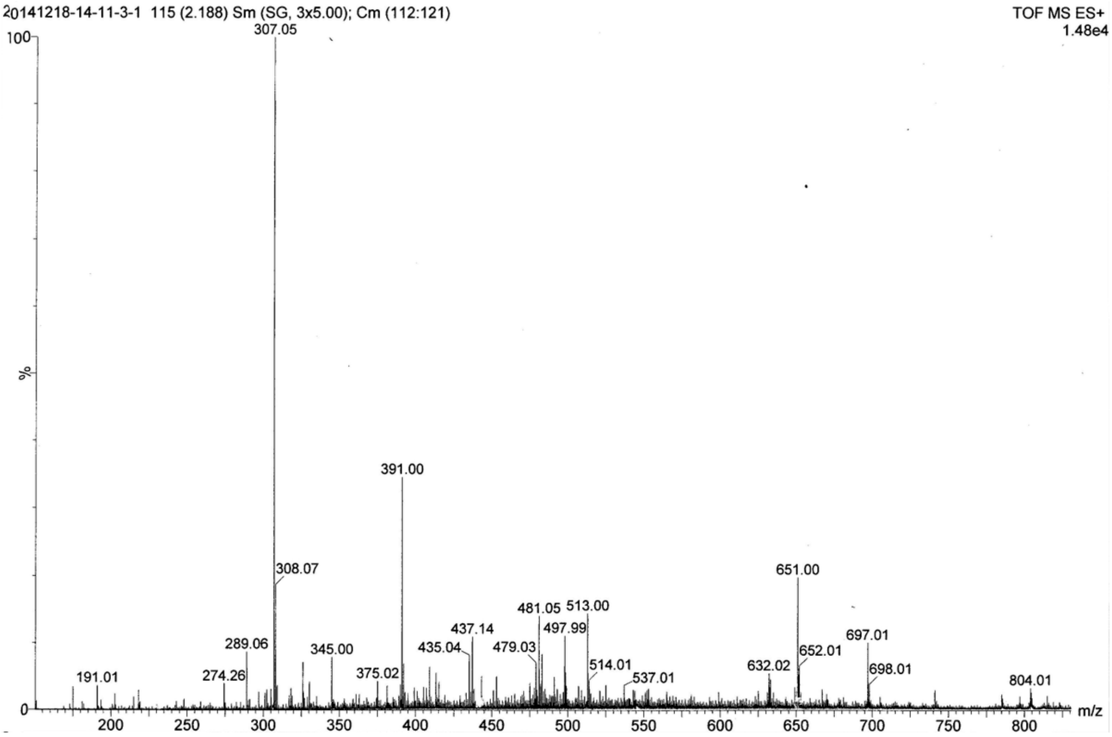

Figure S9. MS spectrum of compound **3**.

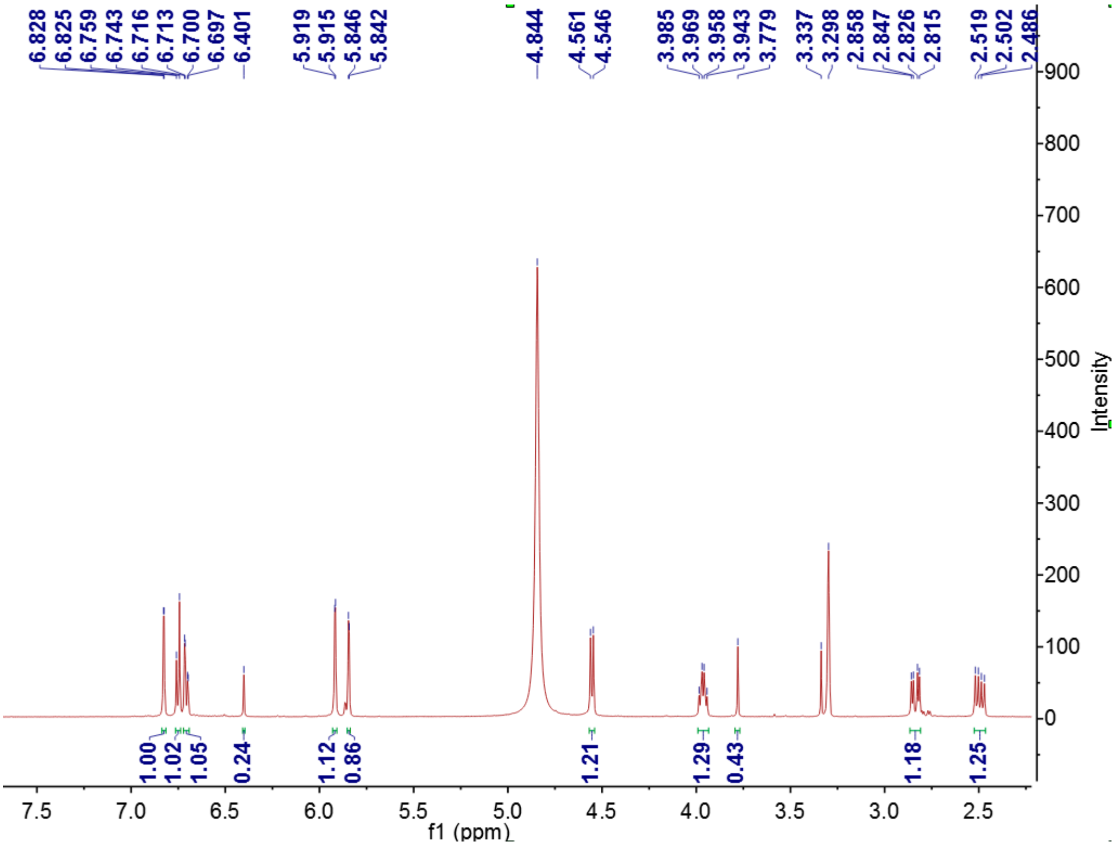

Figure S10. ^{1}H NMR (500 MHz, CD_3OD) spectrum of compound **4**.

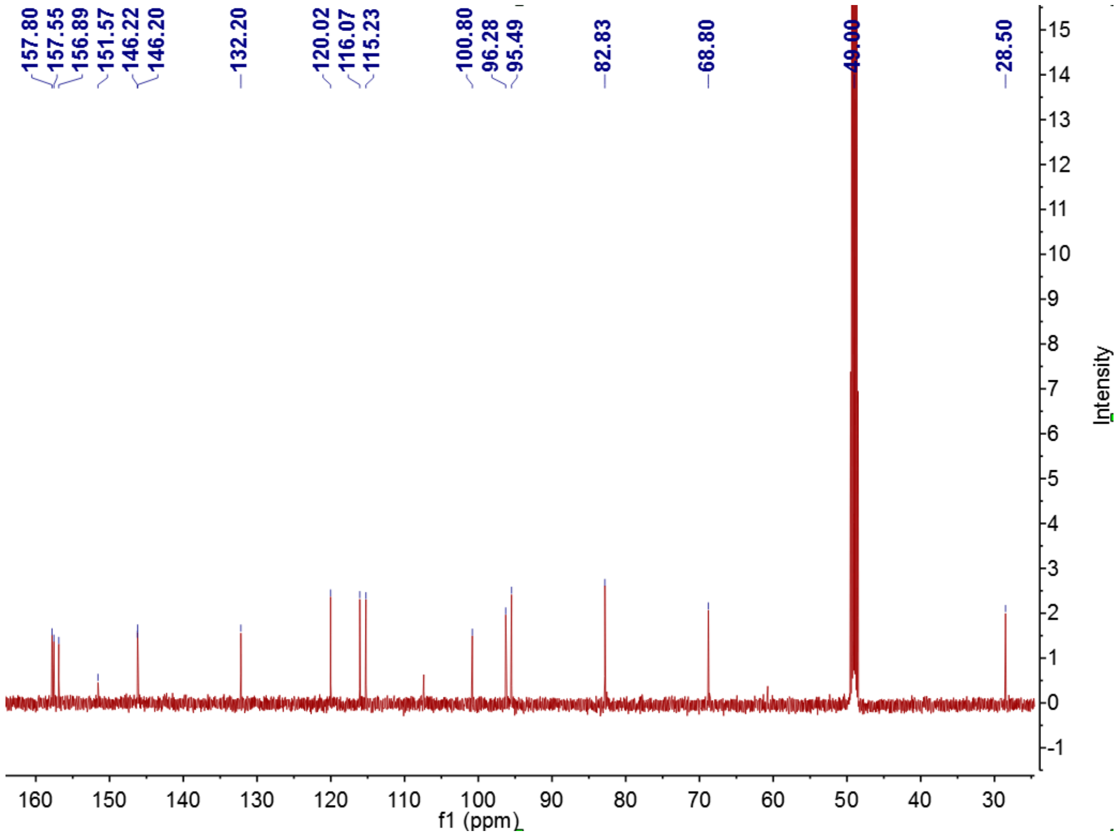

Figure S11. ^{13}C NMR (125 MHz, CD_3OD) spectrum of compound **4**.

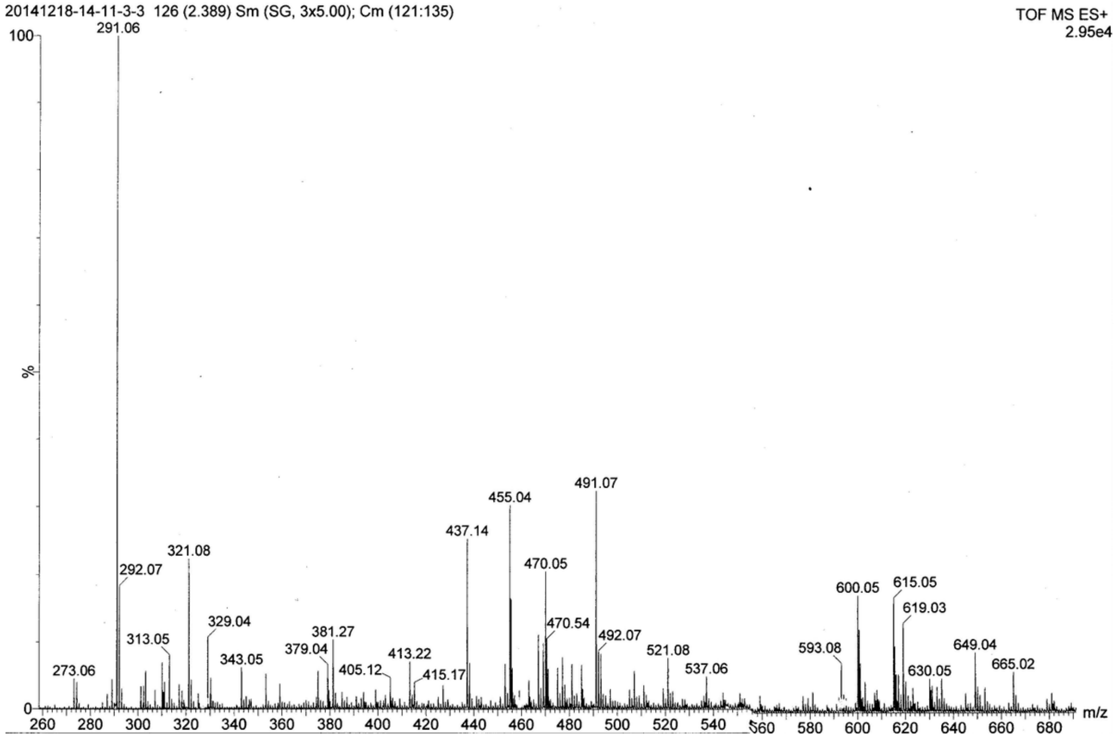

Figure S12. MS spectrum of compound **4**.

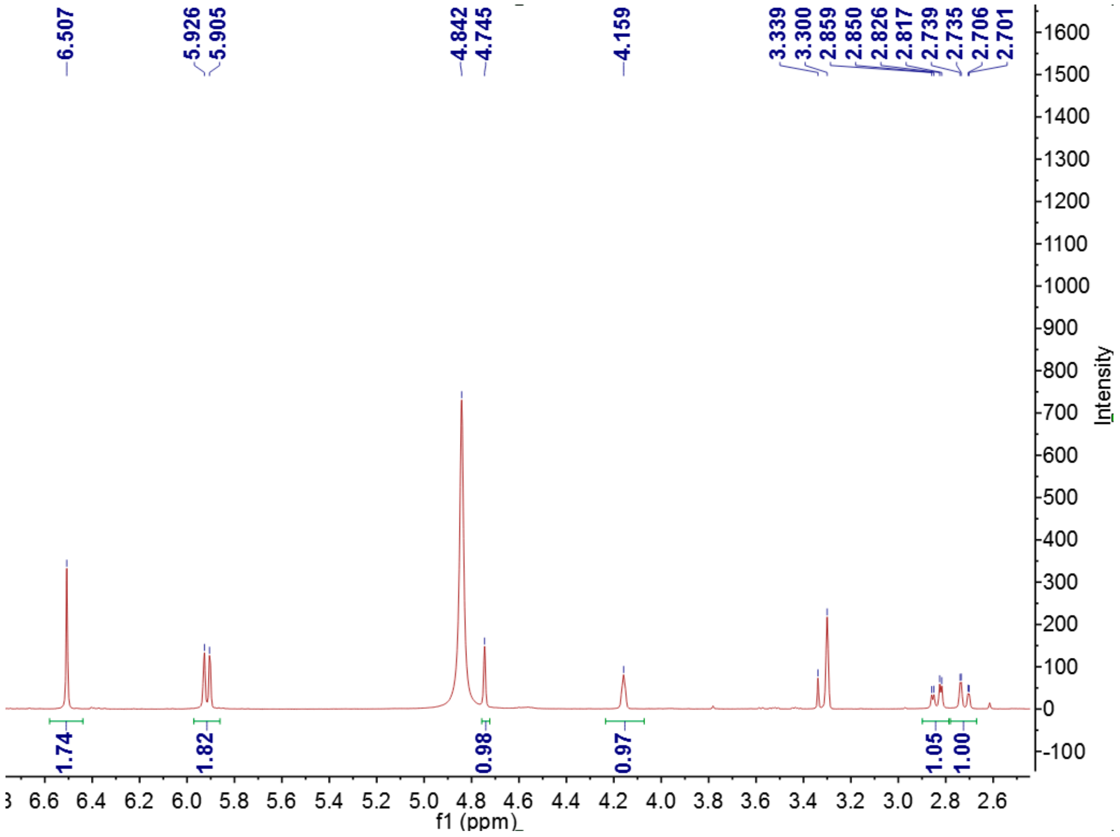

Figure S13. ^{1}H NMR (500 MHz, CD_3OD) spectrum of compound **5**.

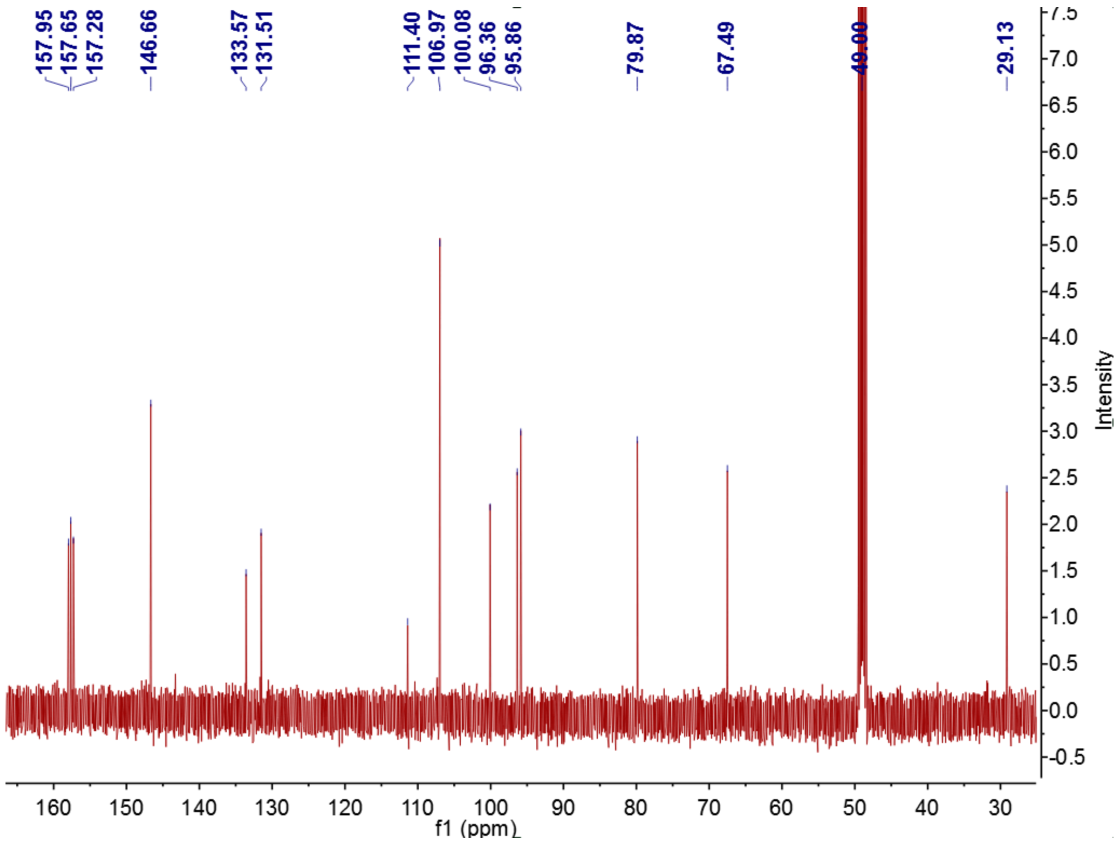

Figure S14. ^{13}C NMR (125 MHz, CD_3OD) spectrum of compound **5**.

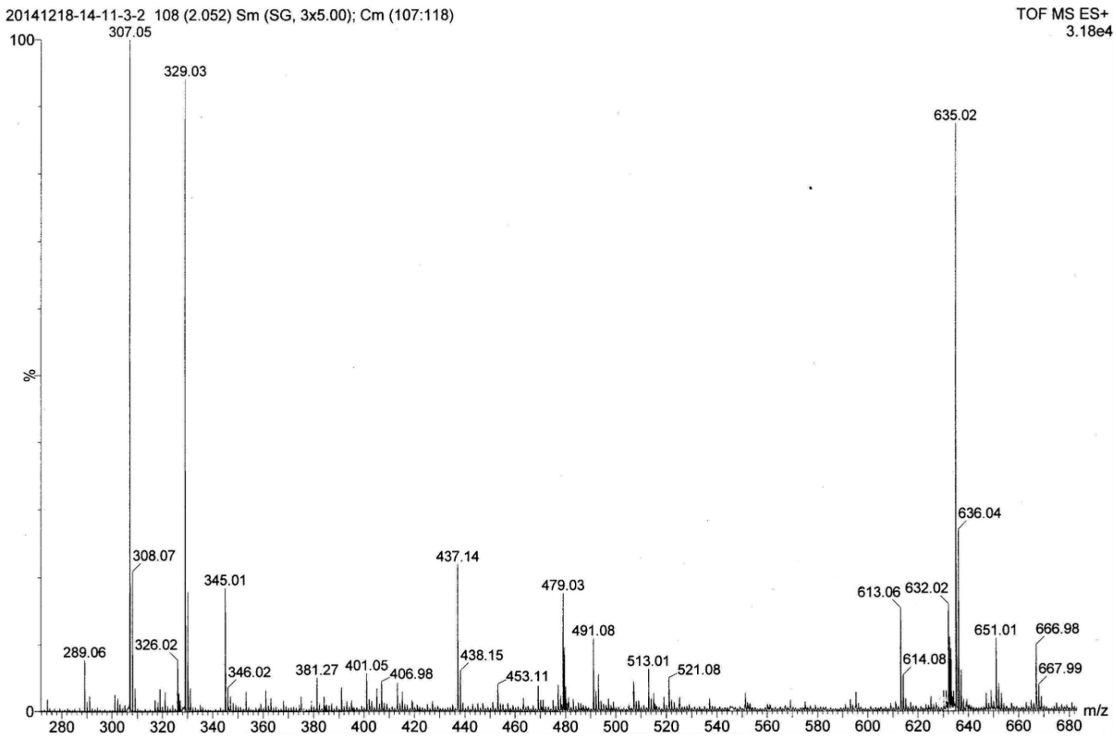

Figure S15. MS spectrum of compound **5**.

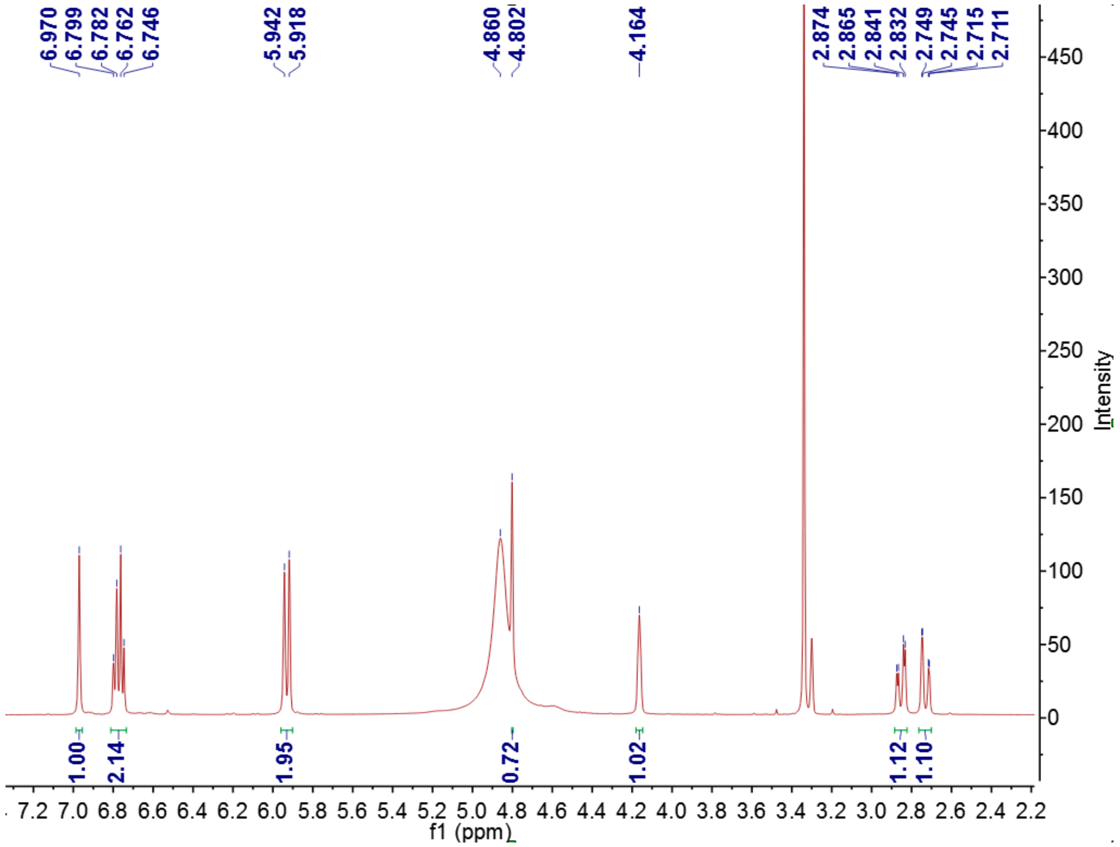

Figure S16. ^{1}H NMR (500 MHz, CD_3OD) spectrum of compound **6**.

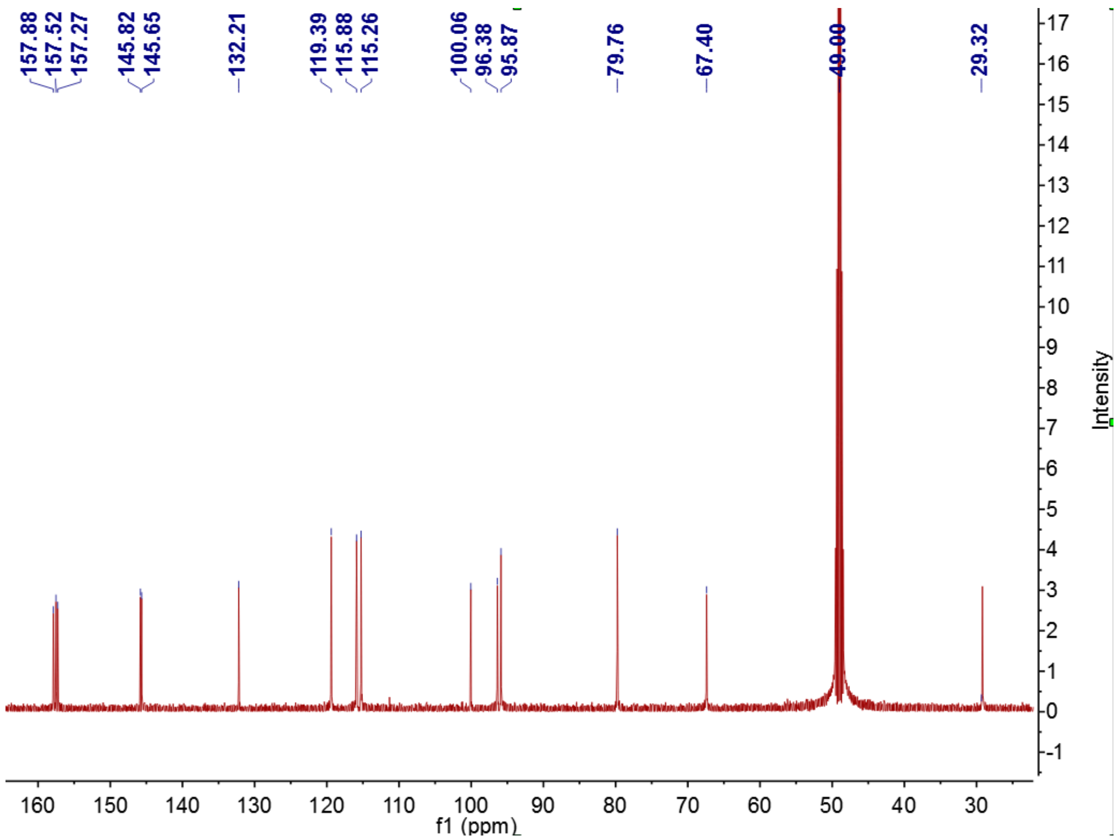

Figure S17. ^{13}C NMR (125 MHz, CD_3OD) spectrum of compound **6**.

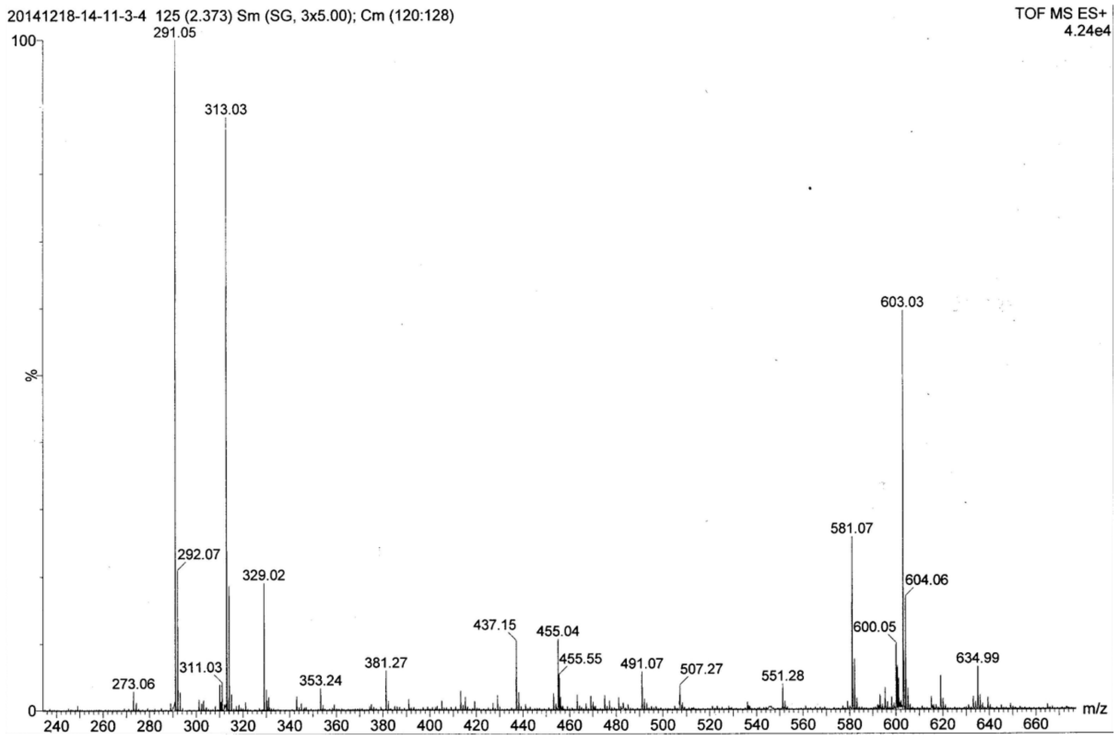

Figure S18. MS spectrum of compound **6**.

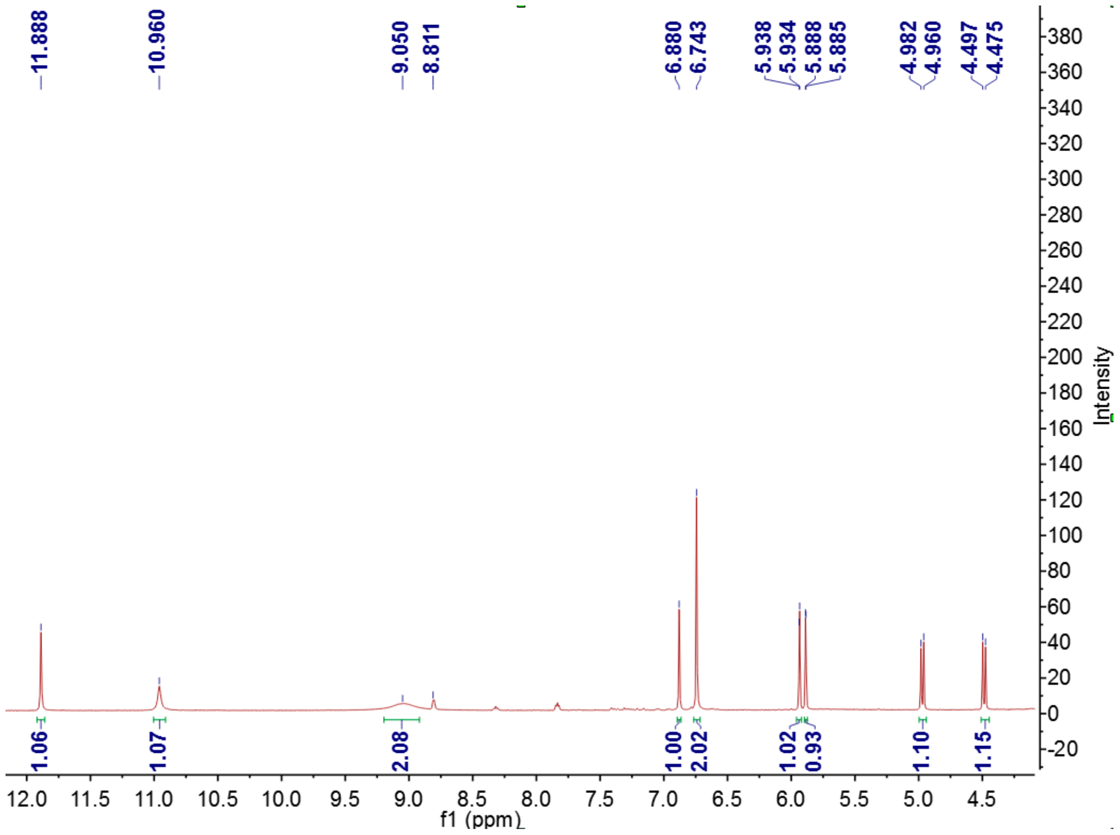

Figure S19. ^{1}H NMR (500 MHz, DMSO-d_6) spectrum of compound **7**.

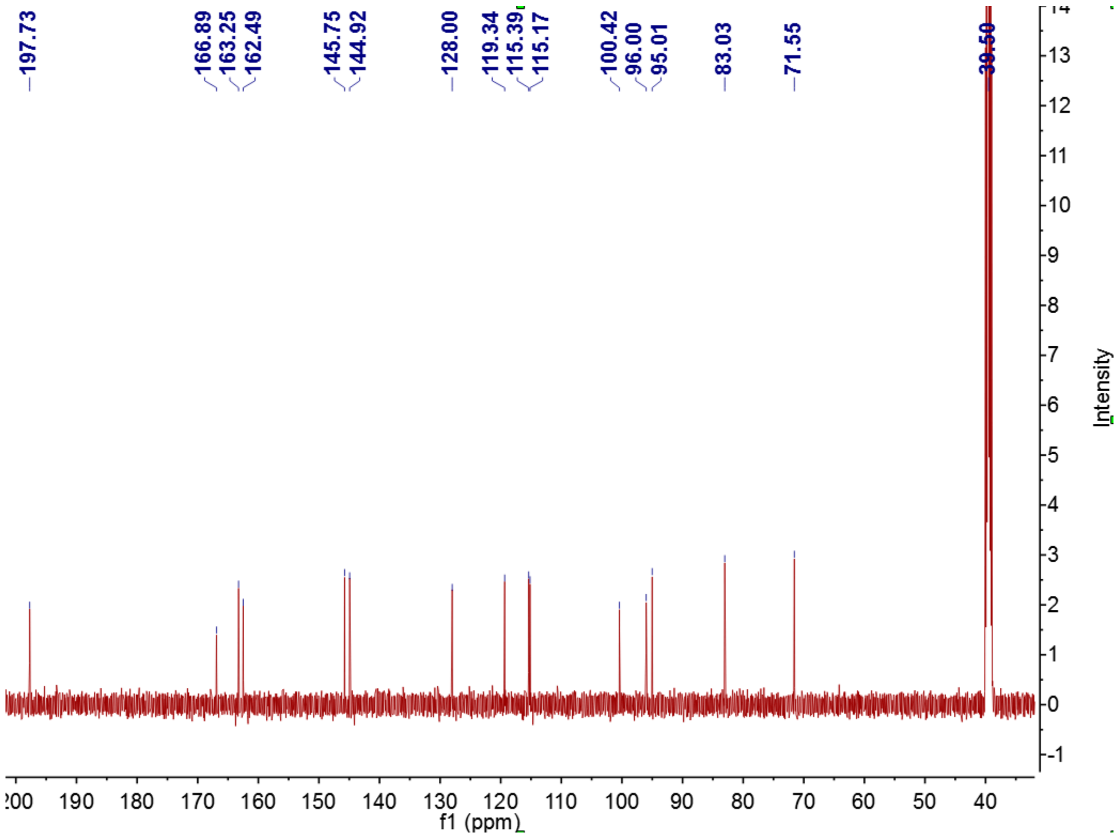

Figure S20. ^{13}C NMR (500 MHz, DMSO-d_6) spectrum of compound **7**.

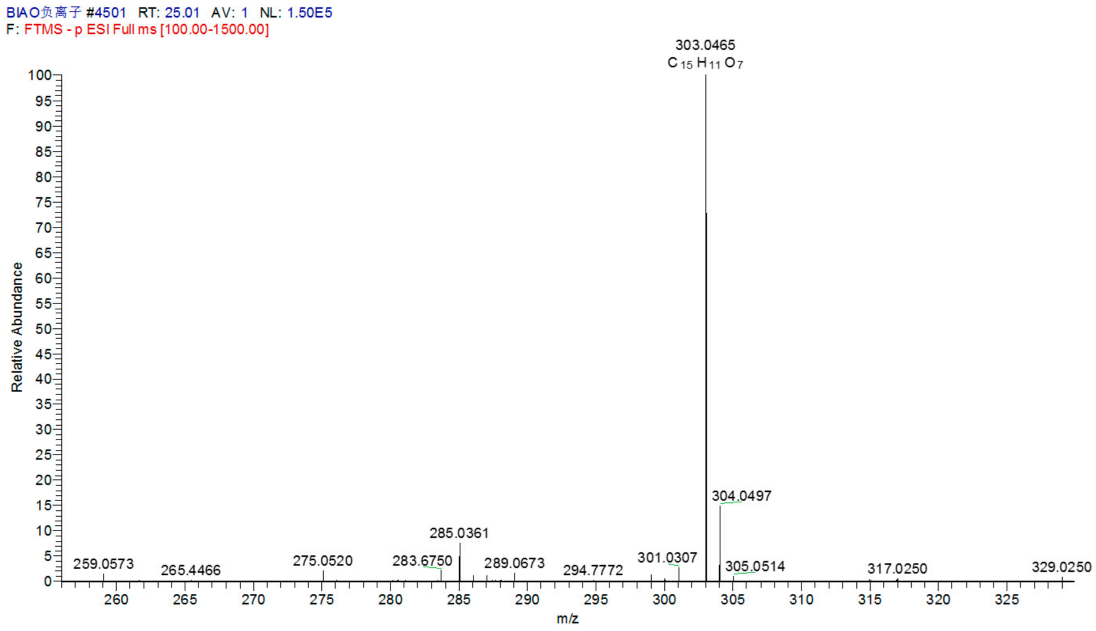

Figure S21. MS spectrum of compound **7**.

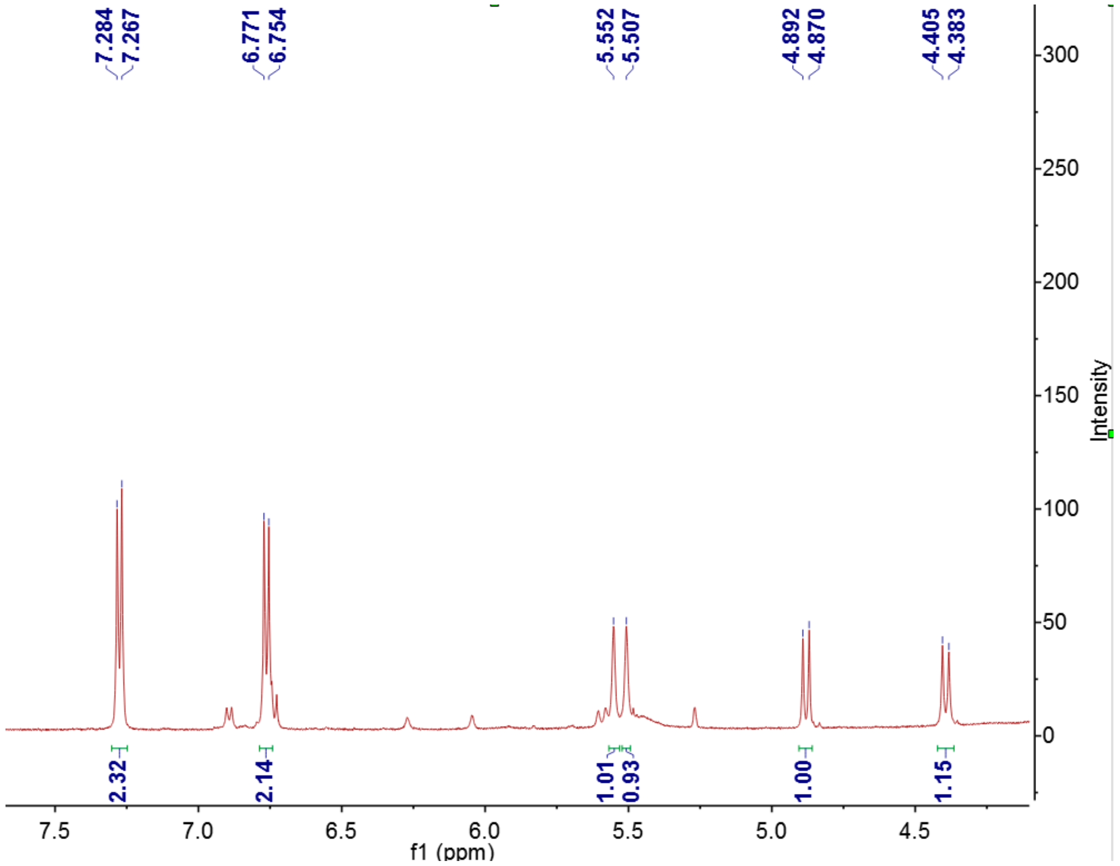

Figure S22. ^{1}H NMR (500 MHz, DMSO-d_6) spectrum of compound **8**.

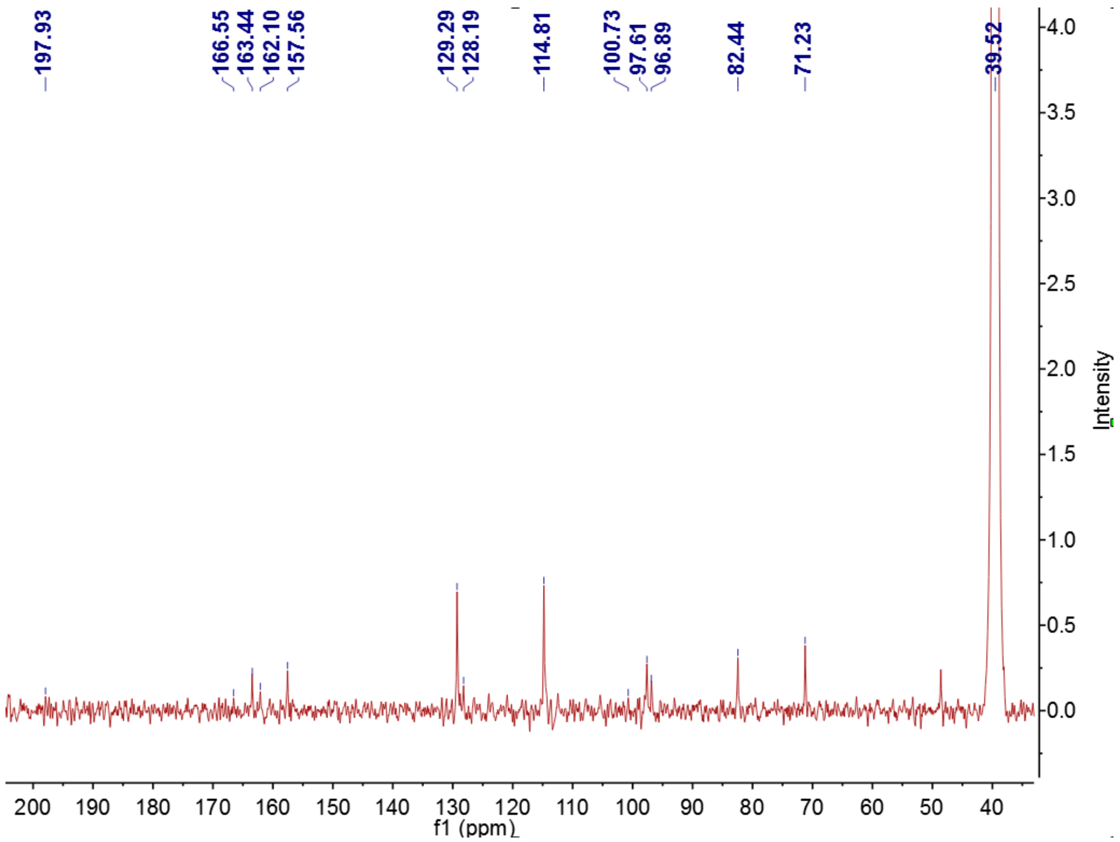

Figure S23. ^{13}C NMR (500 MHz, DMSO-d_6) spectrum of compound **8**.

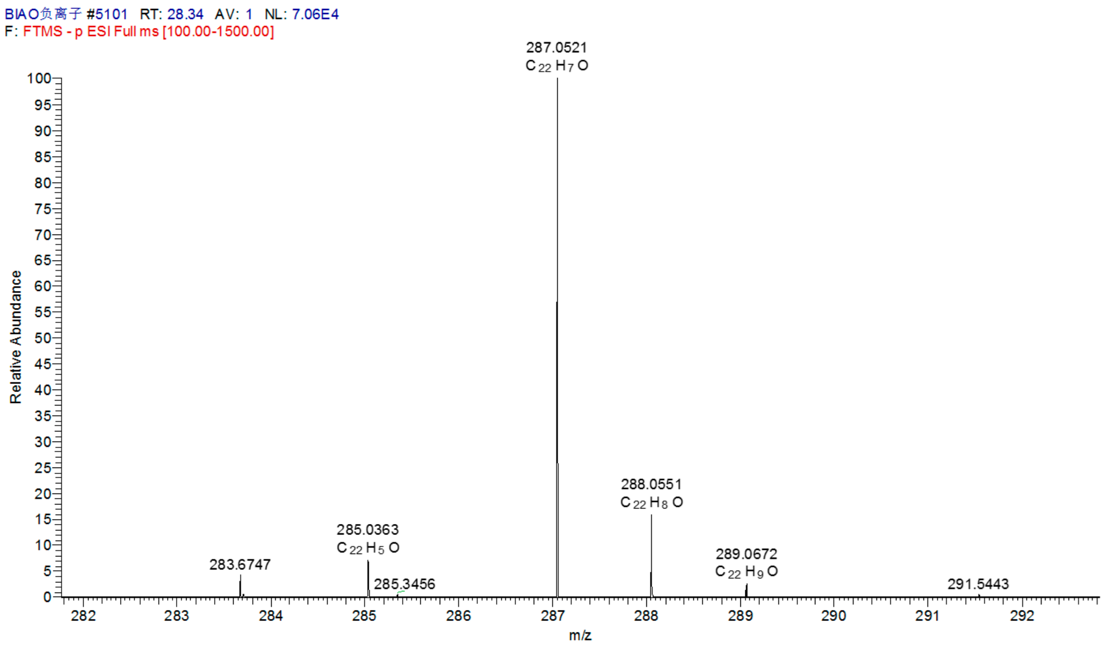

Figure S24. MS spectrum of compound **8**.

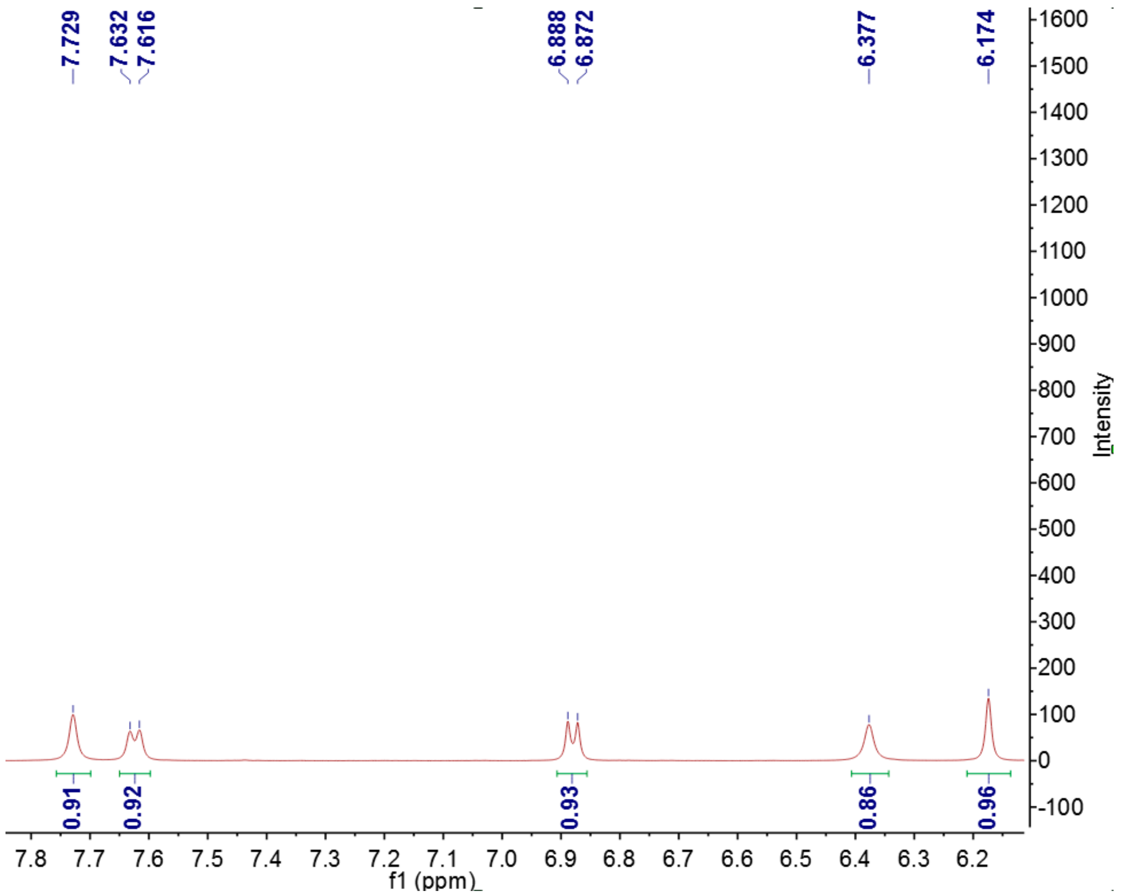

Figure S25. ^{1}H NMR (500 MHz, CD_3OD) spectrum of compound **9**.

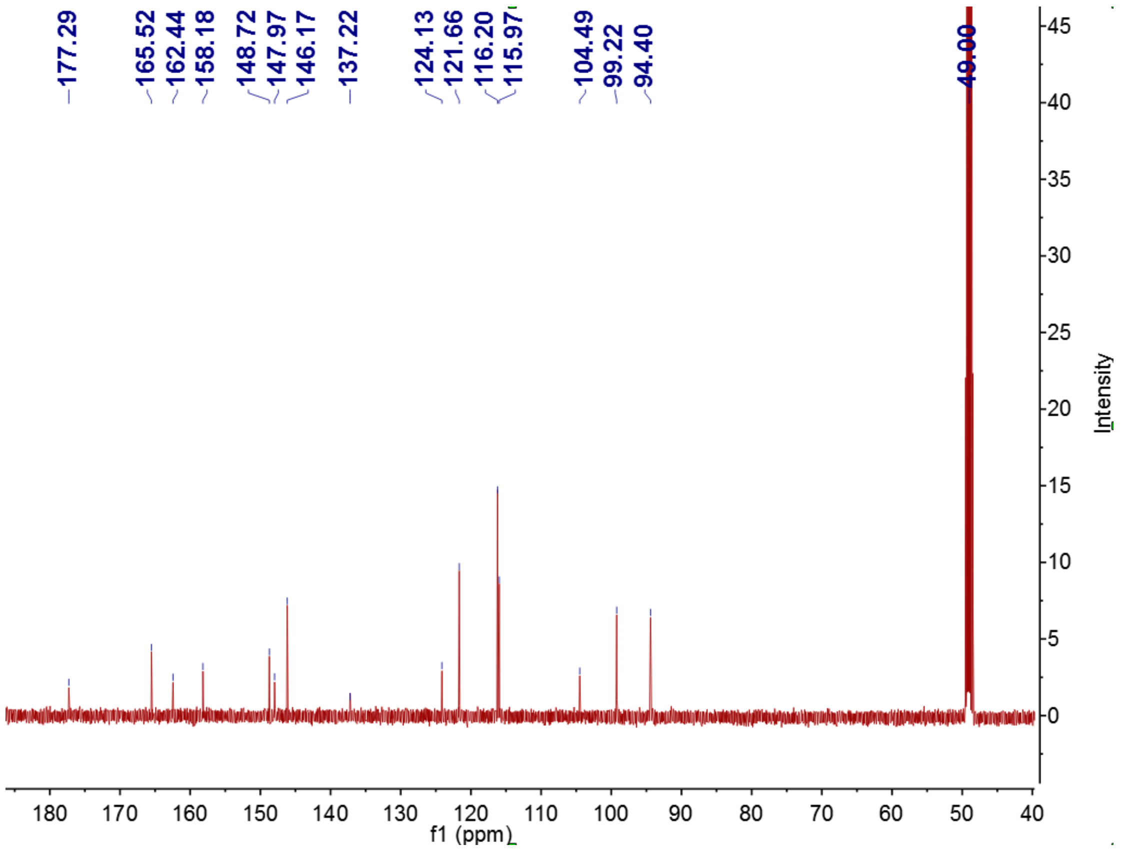

Figure S26. ^{13}C NMR (500 MHz, CD_3OD) spectrum of compound **9**.

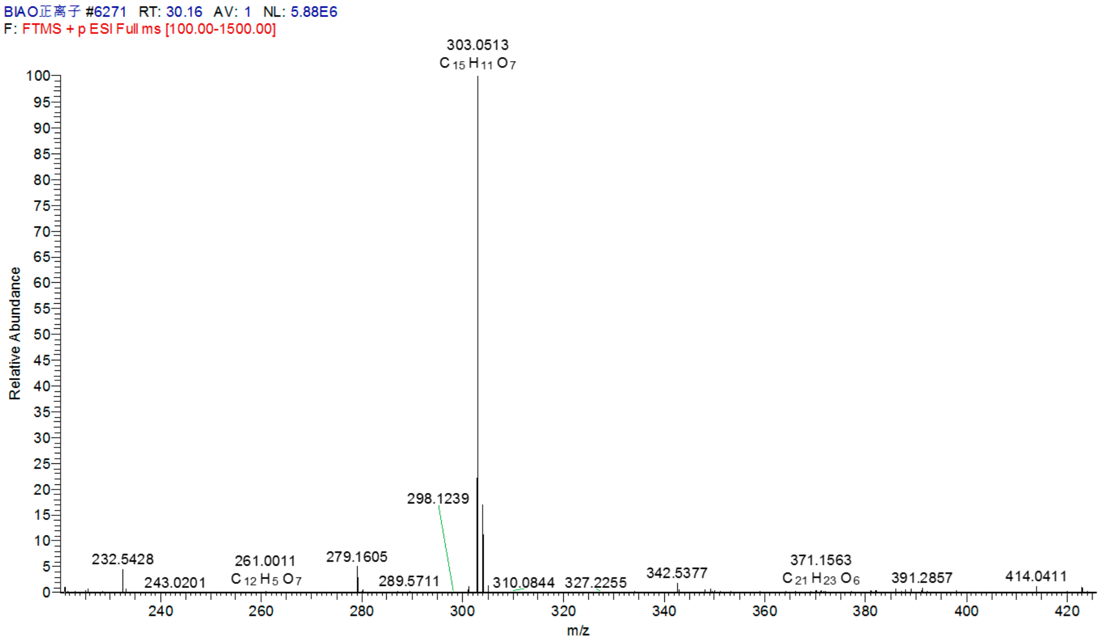

Figure S27. MS spectrum of compound **9**.

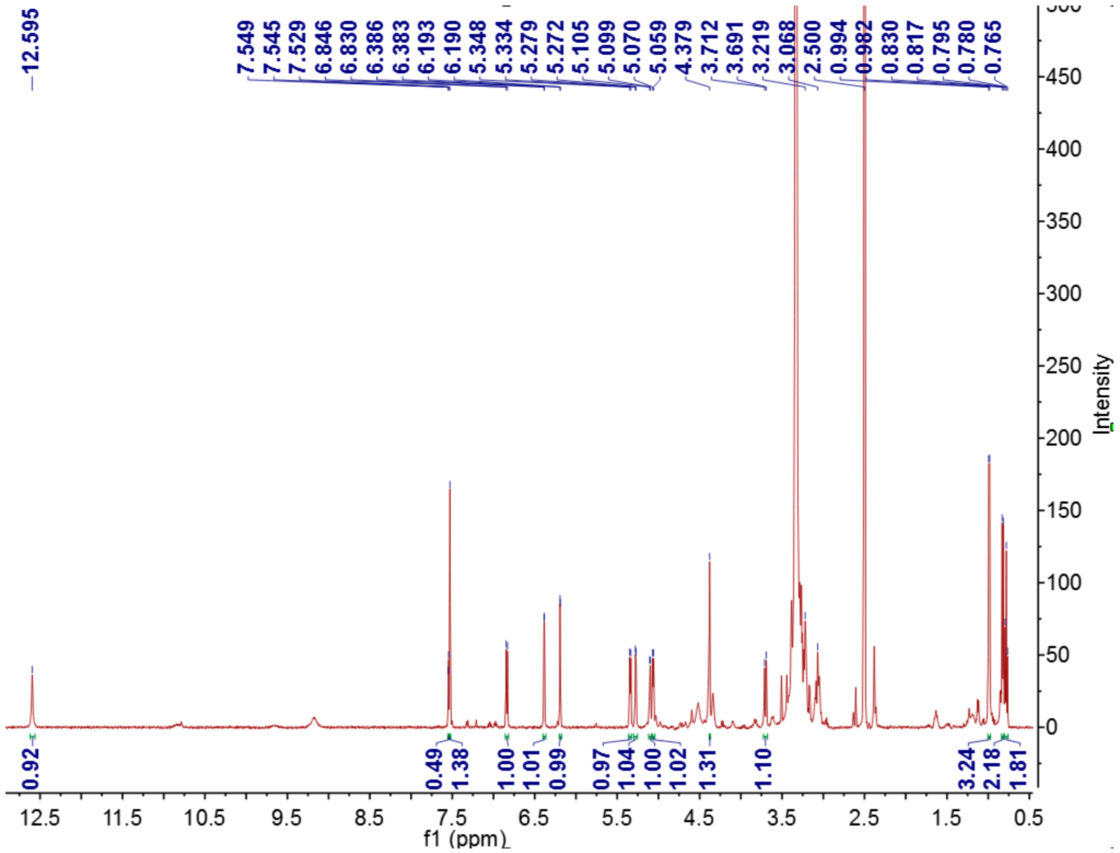

Figure S28. ^{1}H NMR (500 MHz, DMSO-d_6) spectrum of compound **10**.

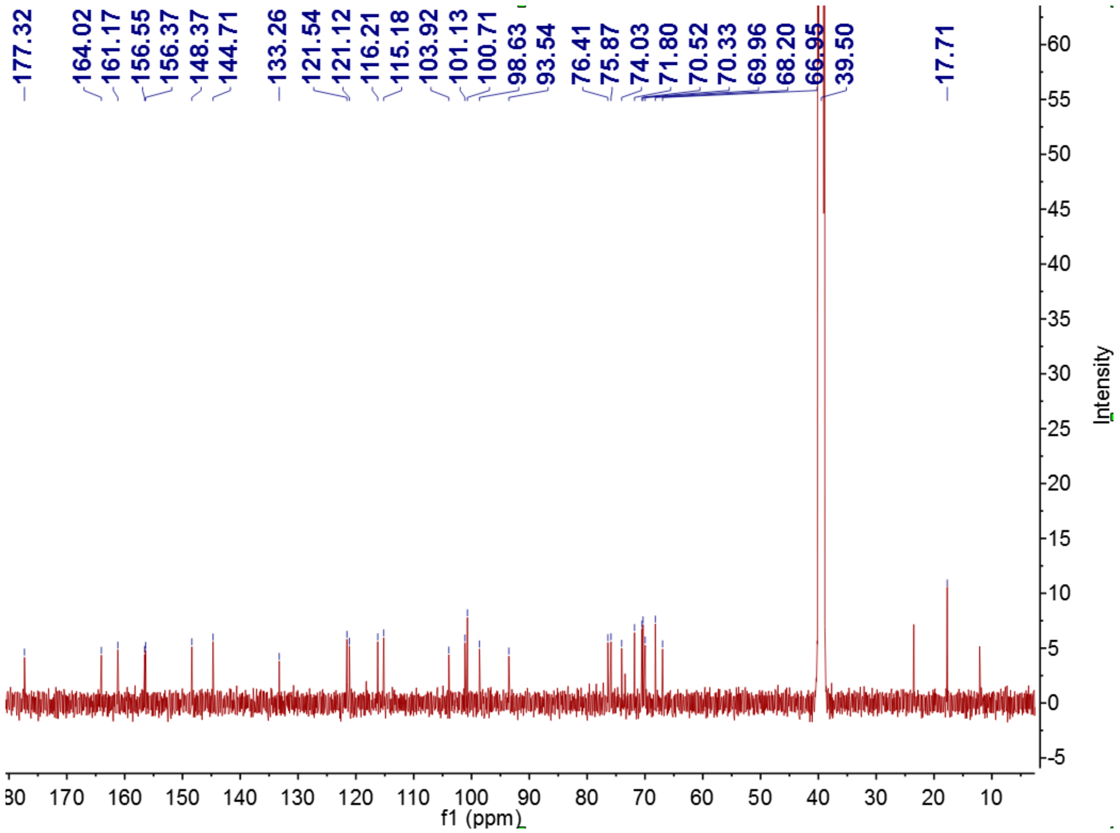

Figure S29. ^{13}C NMR (500 MHz, DMSO-d_6) spectrum of compound **10**.

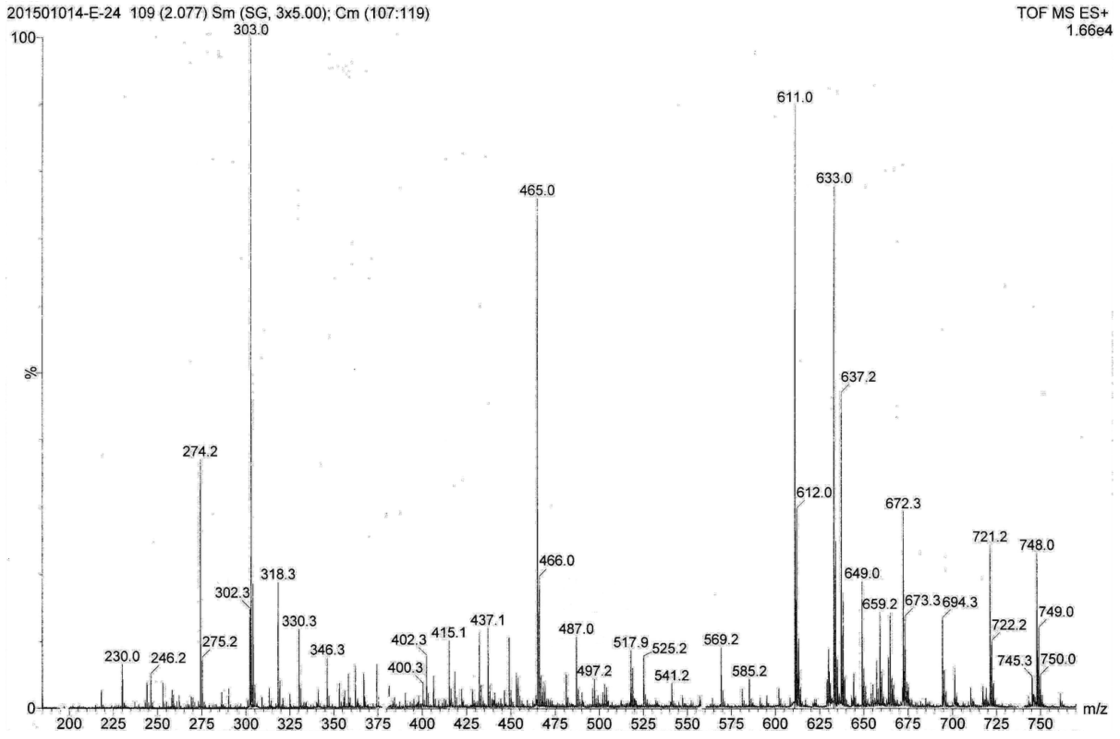

Figure S30. MS spectrum of compound **10**.

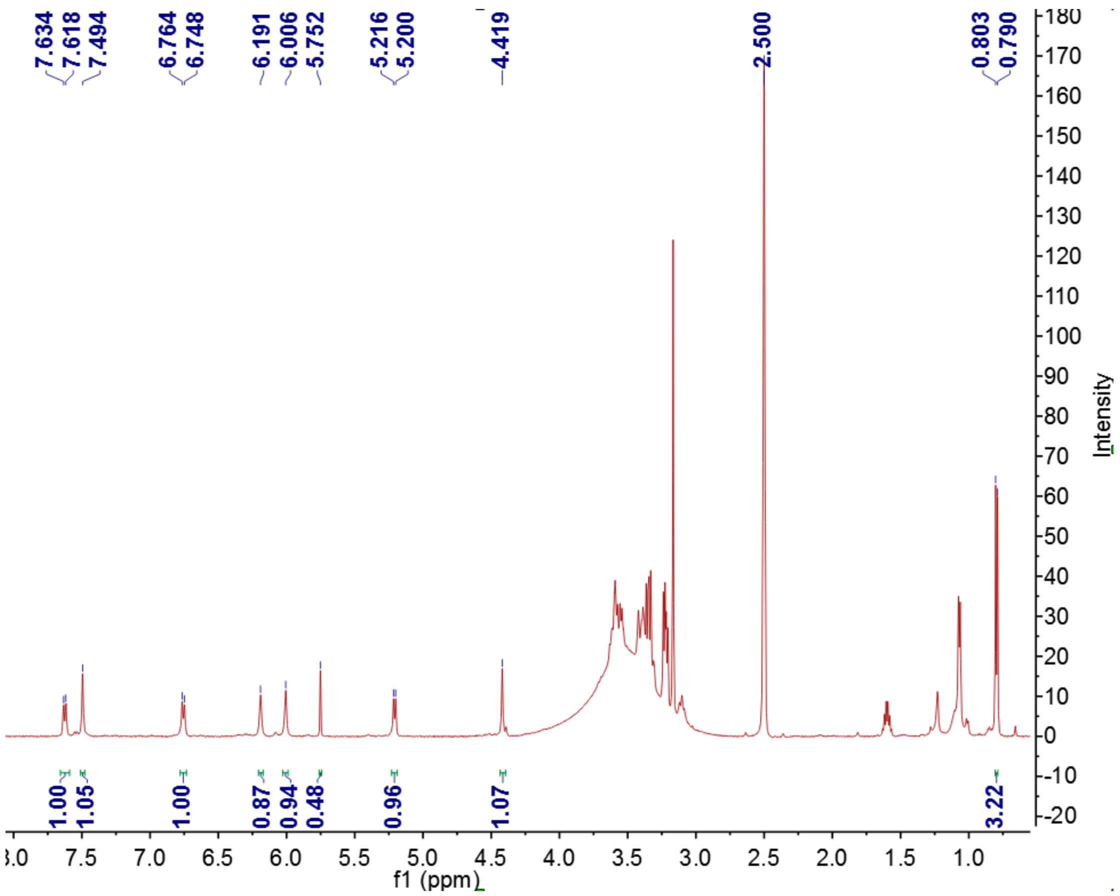

Figure S31. ^{1}H NMR (500 MHz, DMSO-d_6) spectrum of compound **11**.

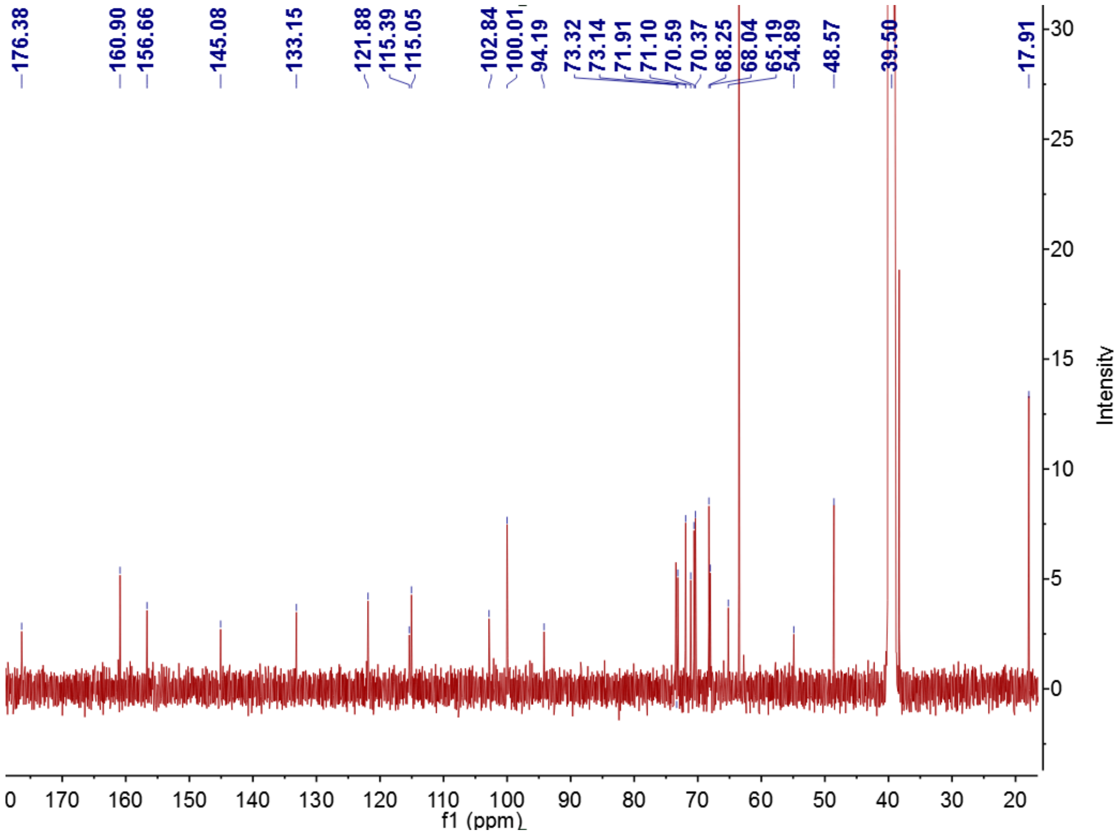

Figure S32. ^{13}C NMR (500 MHz, DMSO-d_6) spectrum of compound **11**.

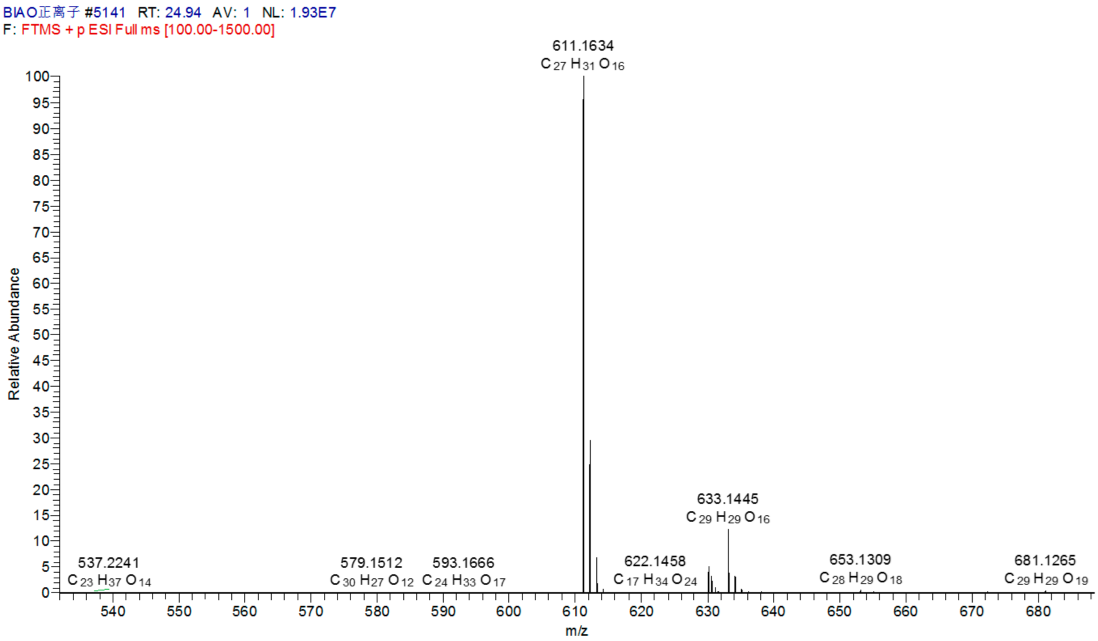

Figure S33. MS spectrum of compound **11**.

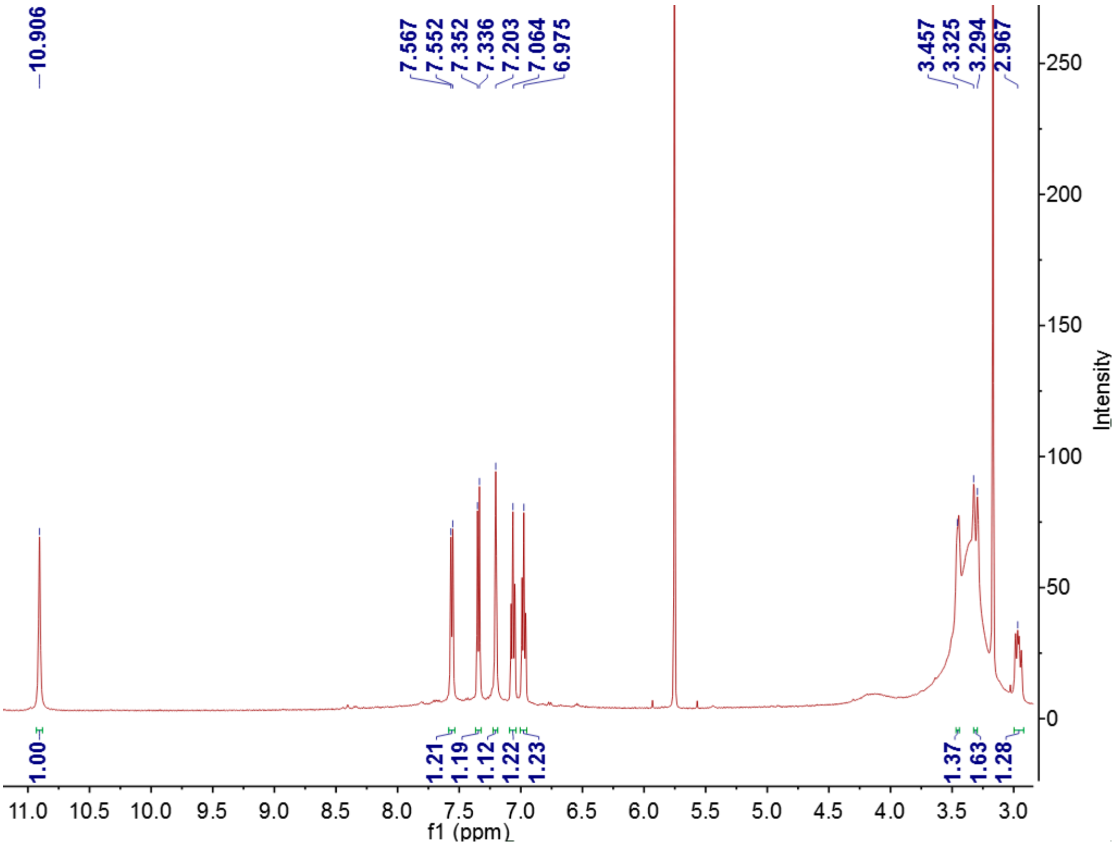

Figure S34. ^{1}H NMR (500 MHz, DMSO-d_6) spectrum of compound **16**.

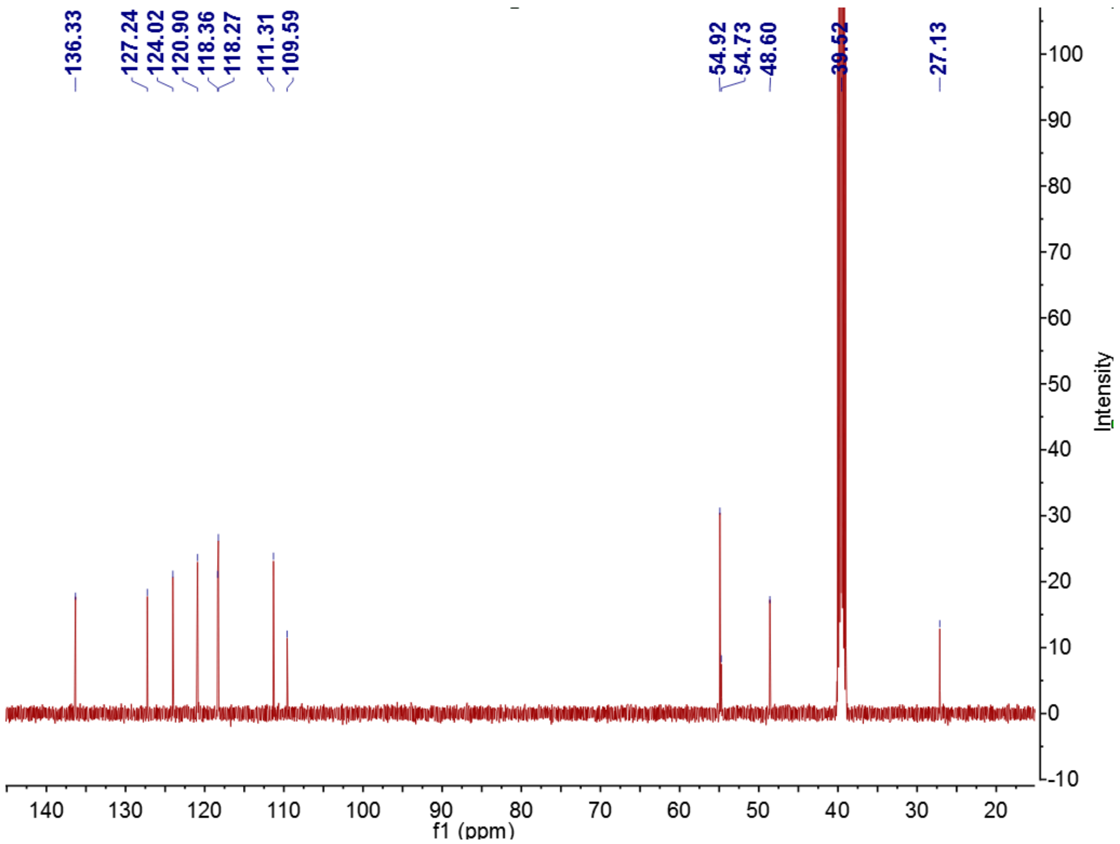

Figure S35. ^{13}C NMR (500 MHz, DMSO-d_6) spectrum of compound **16**.

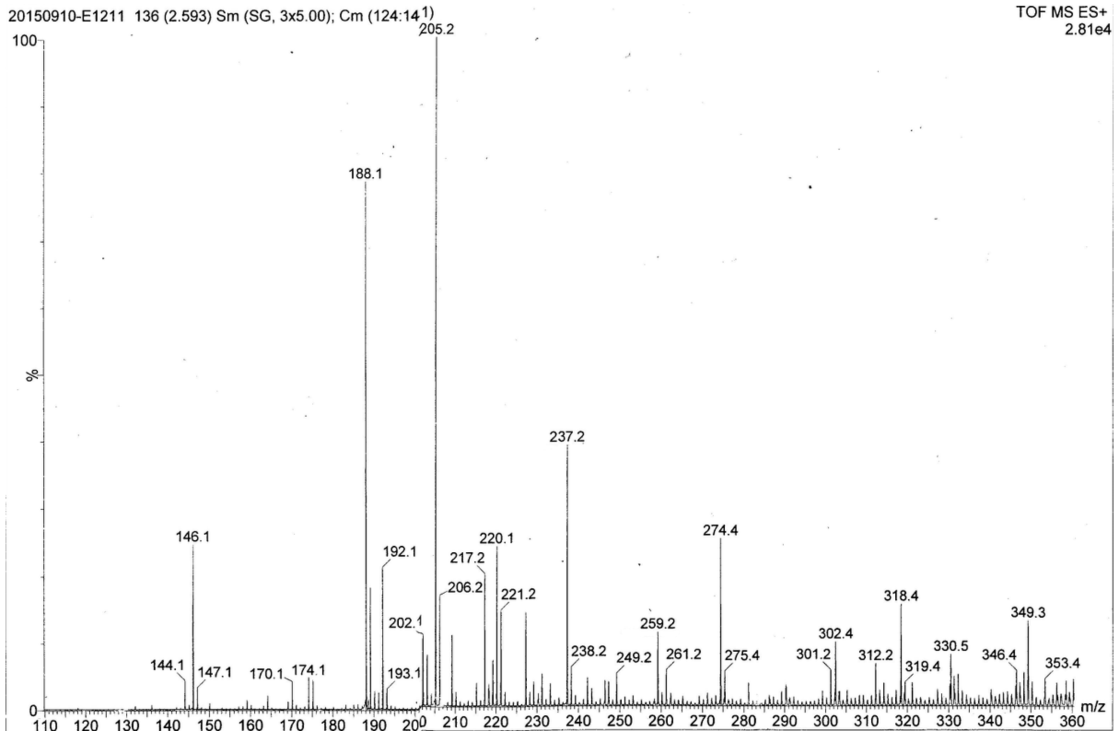

Figure S36. MS spectrum of compound **16**.

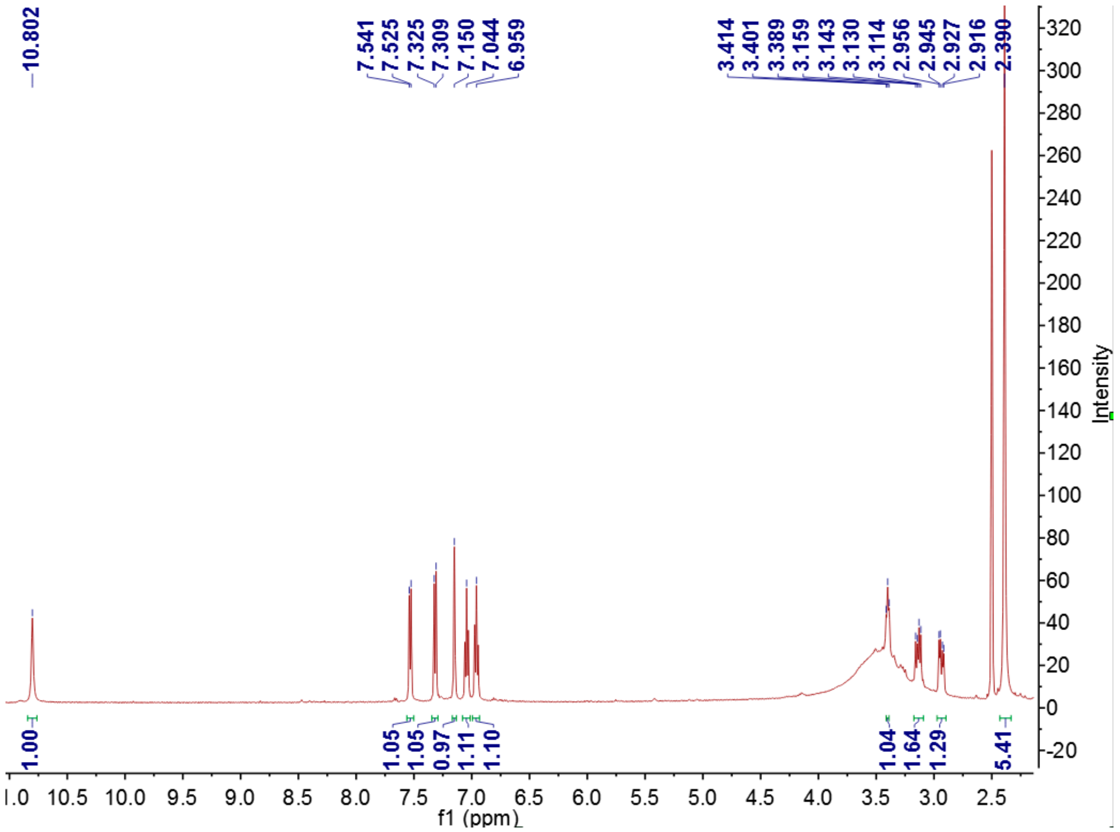

Figure S37. ^{1}H NMR (500 MHz, DMSO-d_6) spectrum of compound **17**.

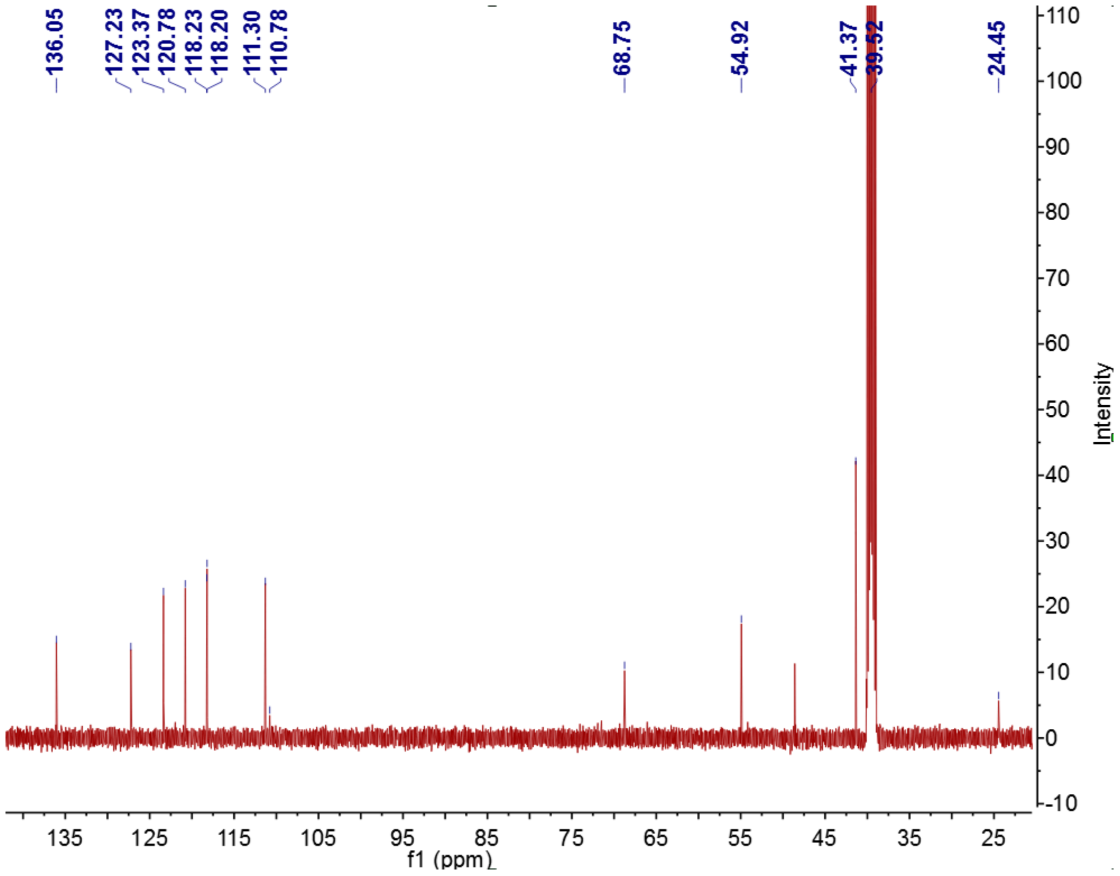

Figure S38. ^{13}C NMR (500 MHz, DMSO-d_6) spectrum of compound **17**.

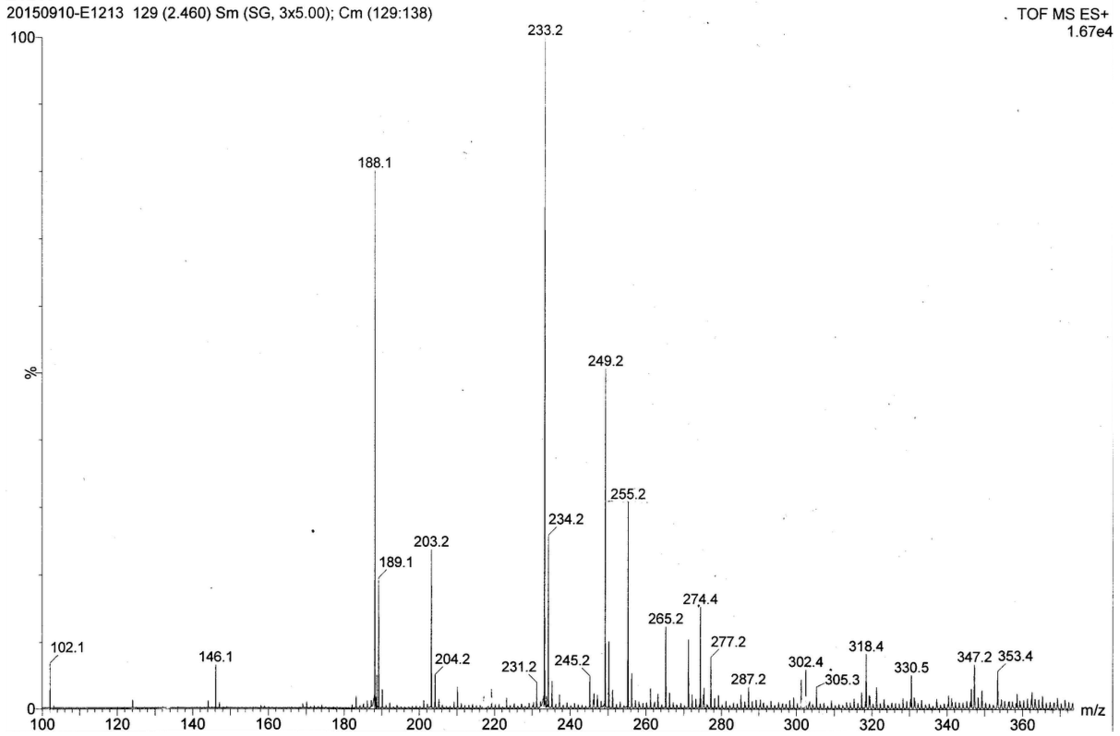

Figure S39. MS spectrum of compound **17**.

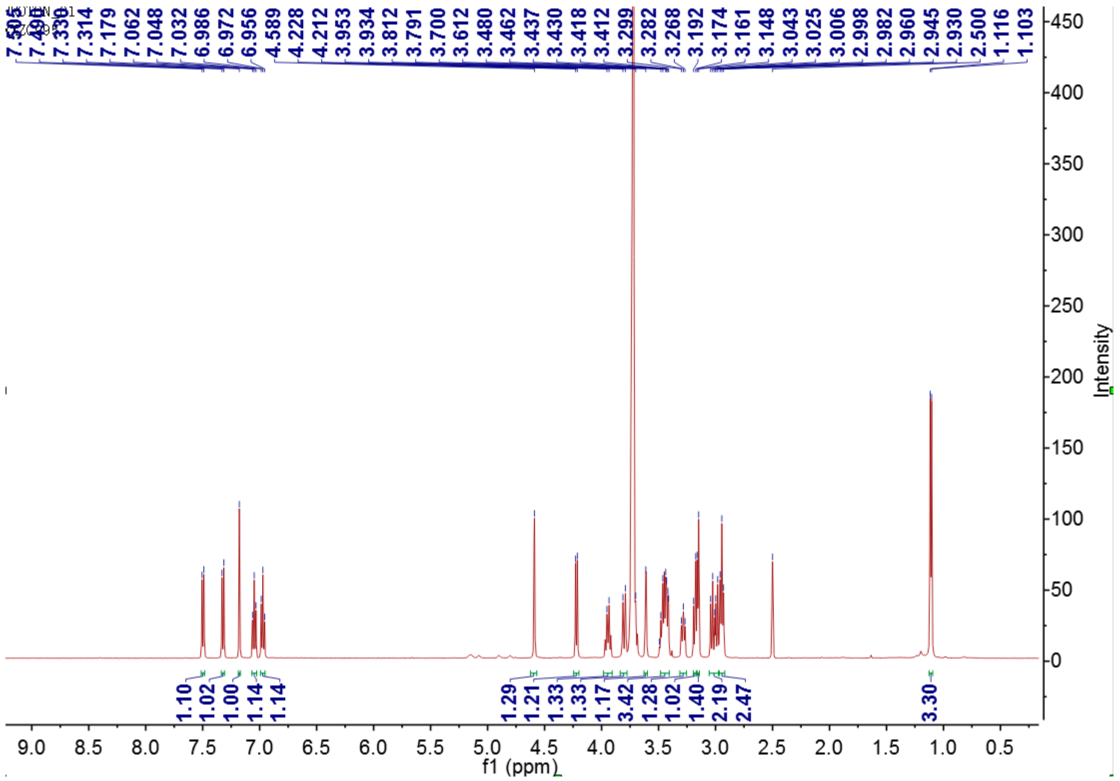

Figure S40. ^{1}H NMR (500 MHz, DMSO-d_6) spectrum of compound **18**.

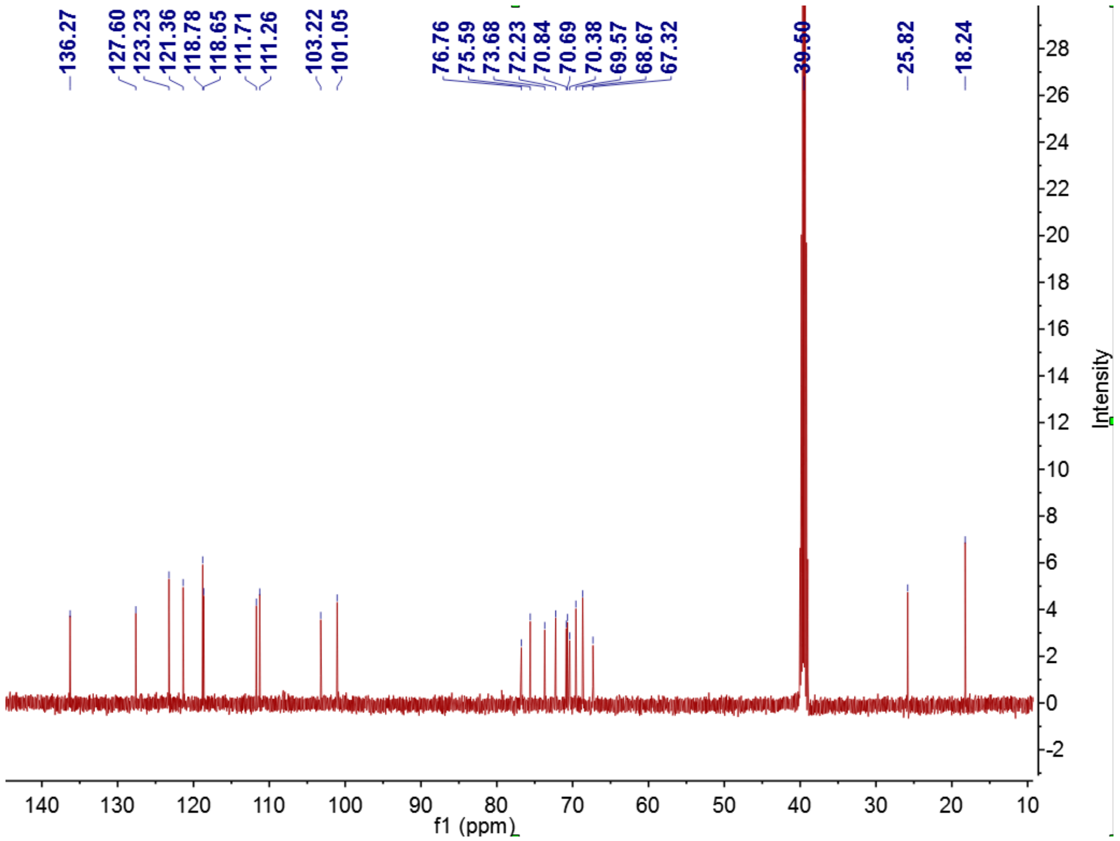

Figure S41. ^{13}C NMR (500 MHz, DMSO-d_6) spectrum of compound **18**.

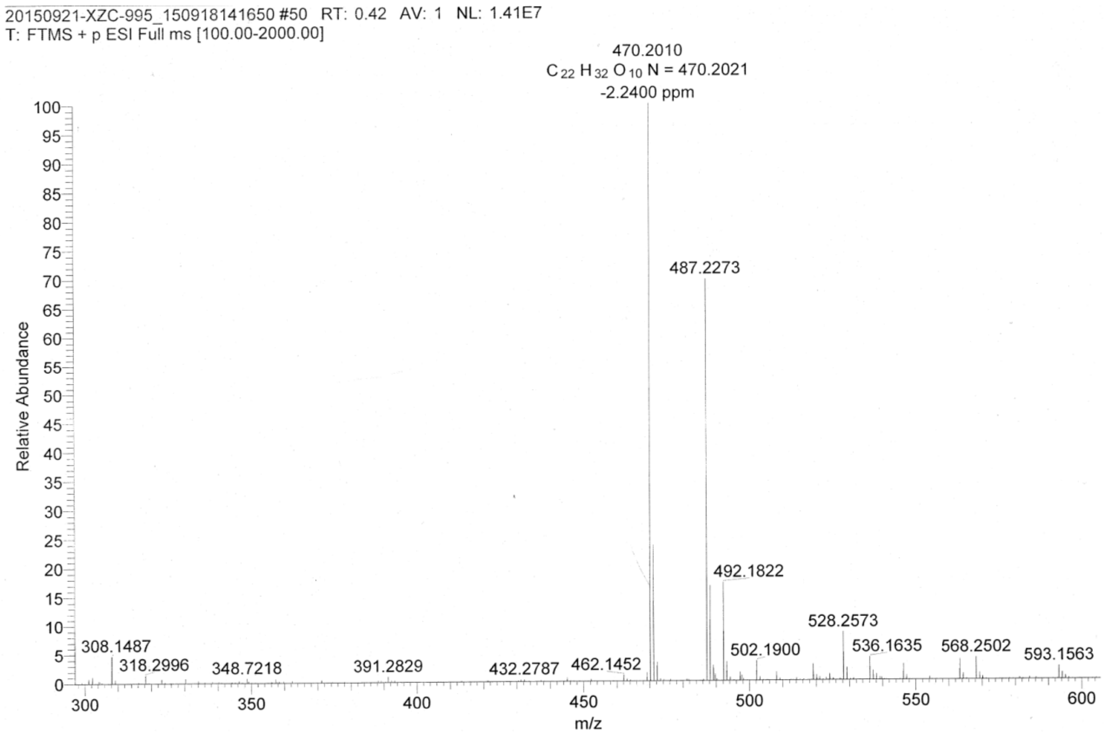

Figure S42. MS spectrum of compound **18**.

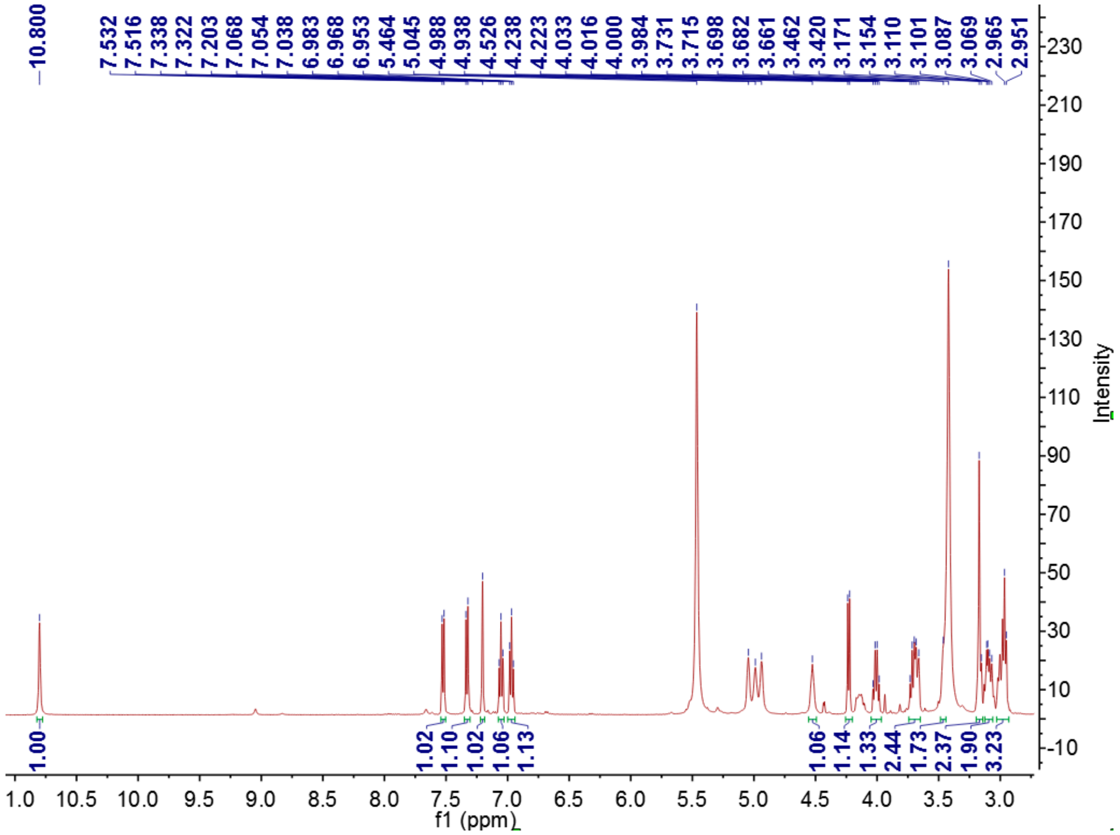

Figure S43. ^{1}H NMR (500 MHz, DMSO-d_6) spectrum of compound **19**.

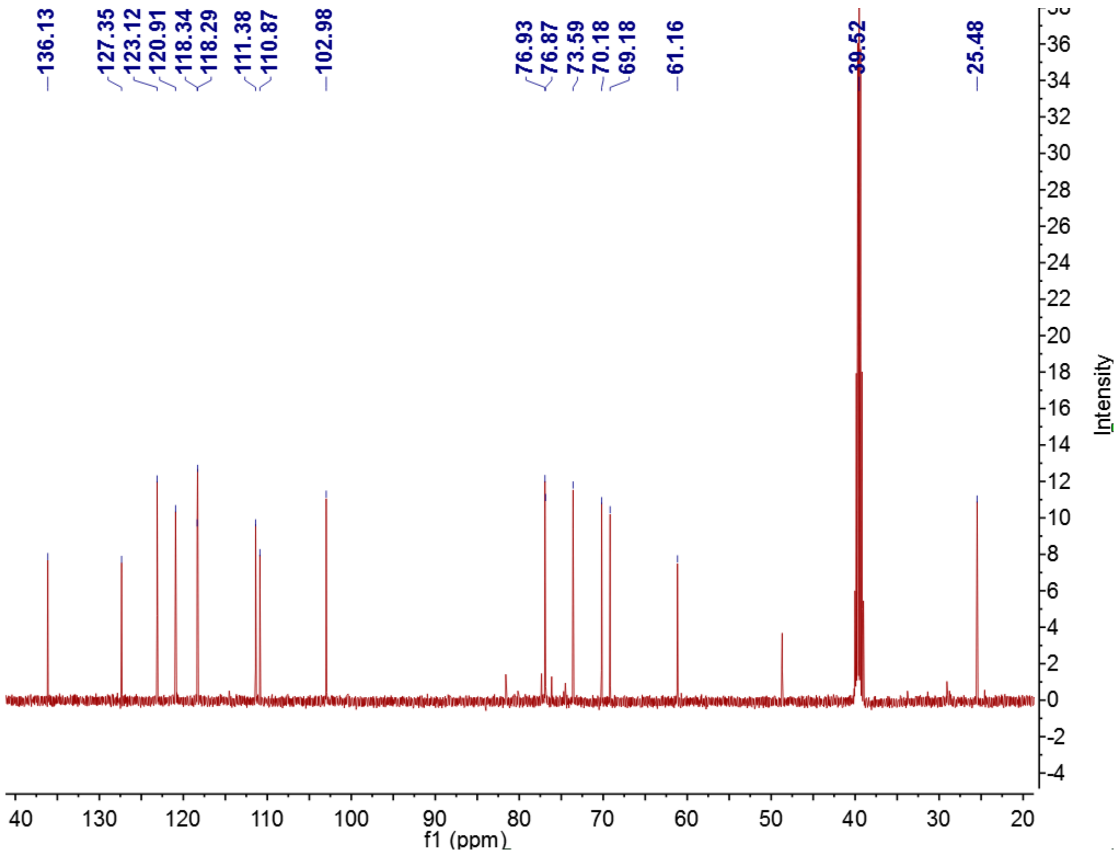

Figure S44. ^{13}C NMR (500 MHz, DMSO-d_6) spectrum of compound **19**.

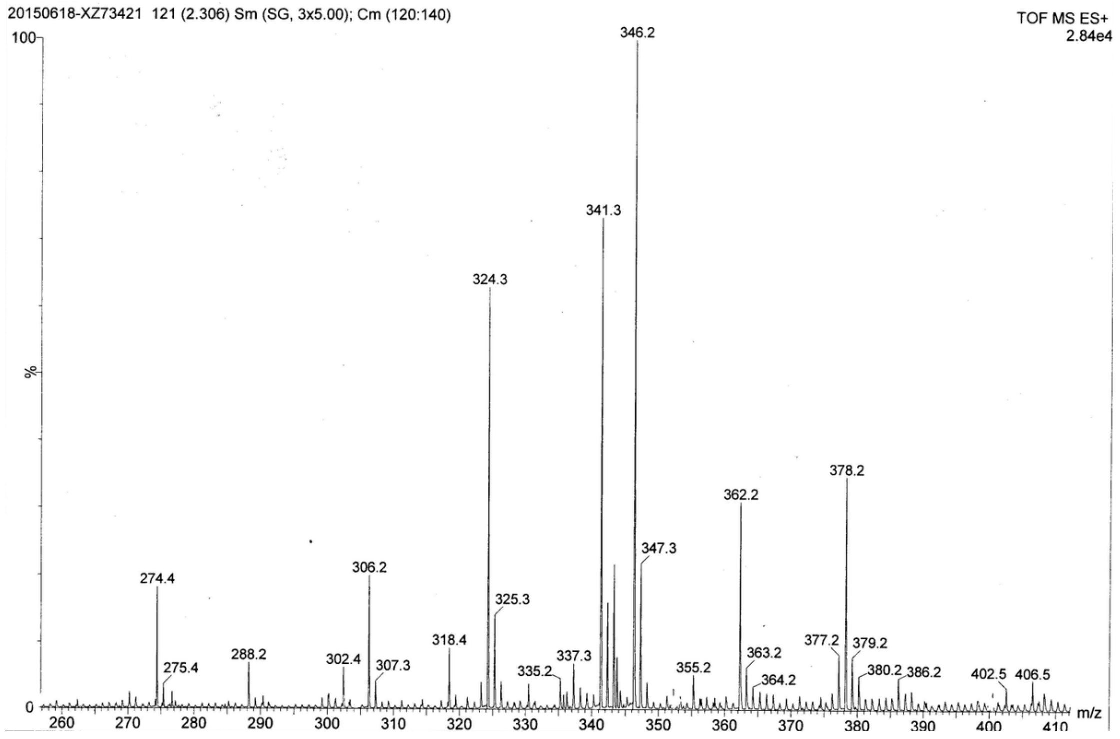

Figure S45. MS spectrum of compound **19**.

The possible fragmentation pathway for compound 2

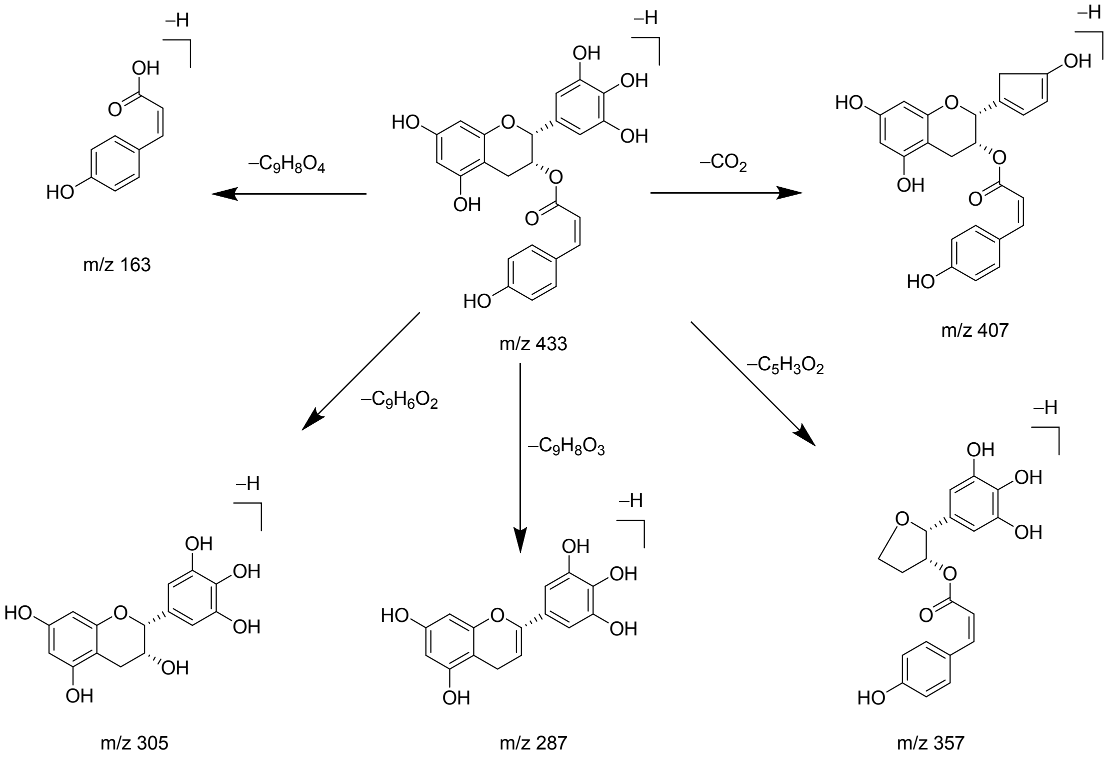

Figure S46. The possible fragmentation pathway for compound **2**.
